# Network Analyses and Data Integration of Proteomics and Metabolomics From Leaves of Two Contrasting Varieties of Sugarcane in Response to Drought

**DOI:** 10.3389/fpls.2019.01524

**Published:** 2019-11-28

**Authors:** Ilara Gabriela Frasson Budzinski, Fabricio Edgar de Moraes, Thais Regiani Cataldi, Lívia Maria Franceschini, Carlos Alberto Labate

**Affiliations:** Laboratório Max Feffer de Genética de Plantas, Departamento de Genética, Escola Superior de Agricultura Luiz de Queiroz, Universidade de São Paulo, Piracicaba, Brazil

**Keywords:** drought, label-free quantitative proteomics, metabolomics, canonical correlation analysis, sugarcane

## Abstract

Uncovering the molecular mechanisms involved in the responses of crops to drought is crucial to understand and enhance drought tolerance mechanisms. Sugarcane (*Saccharum* spp.) is an important commercial crop cultivated mainly in tropical and subtropical areas for sucrose and ethanol production. Usually, drought tolerance has been investigated by single omics analysis (e.g. global transcripts identification). Here we combine label-free quantitative proteomics and metabolomics data (GC-TOF-MS), using a network-based approach, to understand how two contrasting commercial varieties of sugarcane, CTC15 (tolerant) and SP90-3414 (susceptible), adjust their leaf metabolism in response to drought. To this aim, we propose the utilization of regularized canonical correlation analysis (rCCA), which is a modification of classical CCA, and explores the linear relationships between two datasets of quantitative variables from the same experimental units, with a threshold set to 0.99. Light curves revealed that after 4 days of drought, the susceptible variety had its photosynthetic capacity already significantly reduced, while the tolerant variety did not show major reduction. Upon 12 days of drought, photosynthesis in the susceptible plants was completely reduced, while the tolerant variety was at a third of its rate under control conditions. Network analysis of proteins and metabolites revealed that different biological process had a stronger impact in each variety (e.g. translation in CTC15, generation of precursor metabolites, response to stress and energy in SP90-3414). Our results provide a reference data set and demonstrate that rCCA can be a powerful tool to infer experimentally metabolite-protein or protein-metabolite associations to understand plant biology.

## Introduction

Understanding how plants adapt to limiting stress conditions, such as drought, is key to improve global food security. Too much or too little water induces different responses in plant gene expression and metabolism, which we still poorly understand. In Brazil, around 9 million ha are used for sugarcane plantations, producing mainly sugar and ethanol. In recent years, considerable drought events during vegetative growth has led to major losses in productivity, prompting the breeding programs to search for drought tolerant genotypes. Such plant materials are particularly useful for the identification of genetic and metabolic mechanisms involved in sugarcane tolerance to drought, by exploring the omics technologies. Single omics analysis often does not provide enough information to understand the biology of an organism. While the integration of multiple omics may give a better understanding of the biological system, as a whole (Montastier et al., 2015). Global network-based approaches authorize multiple datasets analyses and carry the advantage of highlighting functionally related pathways and biological entities of potential relevance, as hubs (Naylor et al., 2008). rCCA can be applied as an exploratory approach that aims exploiting the linear relationships between two datasets of quantitative variables from the same experimental units (Gonzalez et al., 2008). These assumptions rely on the fact that similar expression patterns across a set of samples are hypothesized to have a functional relationship (Lee et al., 2004). In the present study, we explored the changes in proteome and metabolome responses of two contrasting sugarcane varieties, submitted to a progressive drought. CTC15 (tolerant) and SP90-3414 (susceptible) were submitted to 12 days of drought regime, under greenhouse conditions. The aim of this work was to identify how the leaf proteome and metabolome in these two contrasting varieties interact, leading to different responses to drought. In parallel, we propose the use of rCCA to explore the correlation between metabolites and proteins identified by Label-free quantitative proteomics and GC-MS-based metabolomics. We chose sugarcane (*Saccharum* spp.) as our model plant because this perennial grass, cultivated in tropical and sub-tropical countries, is the world’s most important sugar crop with a high capacity to store sucrose in its culms, as a primary energy source (D’Hont et al., 2008). It has become an important bioenergy source and is classified among the most important crop for first and second-generation ethanol production (Heringer et al., 2015; Hoang et al., 2015). During sugarcane vegetative growth, drought may lead to significant decreases in productivity and yield (Zingaretti et al., 2012). Besides, drought impacts also in membrane integrity, pigment content, osmotic adjustment, water relations, photosynthetic activity (Benjamin and Nielsen, 2006), limiting the areas suitable to sugarcane cultivation. Understanding the molecular mechanisms involved in drought tolerance is a key step to develop sugarcane varieties capable to use water efficiently. Such approach becomes highly significant when one considers the fact that sugarcane has one of the most complex plant genomes, with a high chromosome number and high degree of aneuploidy (D’Hont et al., 1996). This chromosomal complexity makes it difficult to obtain improvements of sugarcane through conventional breeding. In the last few years some publications reported advances in the study of sugarcane responses to drought, using different strategies such as differential gene expression (Vargas et al., 2014; Vantini et al., 2015; Li et al., 2016), sRNA regulation (Thiebaut et al., 2014), morphological and physiological analysis (Cia et al., 2012; Zhao et al., 2013; Jain et al., 2015) and proteomics by two-dimensional gel electrophoresis (Jangpromma et al., 2010; Zhou et al., 2012; Almeida et al., 2013; Khueychai et al., 2015). To our knowledge this is the first exploratory analysis applying rCCA to associate metabolite and protein data in sugarcane response to drought. These omics approaches provide the opportunity to evaluate cellular behaviors from a multi-level perspective and enhance our understanding of sugarcane biology.

## Methods

### Plant Material and Experimental Conditions

To study changes in protein and metabolite profiles in response to drought, two sugarcane varieties were used: CTC15, previously reported as tolerant to drought (Graça et al., 2010; Kido et al., 2012; Silva et al., 2013; Thiebaut et al., 2014), and SP90-3414 susceptible to drought (Cia et al., 2012; Kido et al., 2012; Silva et al., 2013). Stalks from both varieties were kindly provided by CTC (Centro de Tecnologia Canavieira, Piracicaba, Brazil). The experiment was conducted in a greenhouse with a temperature range of 28 °C ± 2 during the day and 22 °C ± 2 during the night. Single stalks were planted, germinated and grown in 50 L pots with a mixture (2:1) of substrate (BasaPlant^®^, Brazil) and vermiculit. Plants were arranged in a completely randomized experimental design, under daily irrigation and the soil moisture was maintained near to 100% of field capacity (FC, equivalent to -234 KPa). After 5 months of growth, leaf gas exchange measurements and samples were obtained from plants at three levels of water availability in the soil: permanently irrigated (PI), 4 and 12 days without irrigation (4 DI and 12 DI, respectively). The soil moisture was maintained in the range of 100%, 40% (-286 KPa), and 25% (-850 KPa) of FC, respectively. Gas exchange rates were determined from five plants of each variety, chosen randomly in the greenhouse. After gas exchange measurements, the top visible dewlap leaves (+1) were detached from nine sugarcane plants for each treatment, frozen immediately in liquid nitrogen and stored at -80°C. For metabolomics each leaf separately was used for metabolite extraction while for proteomics, each three leaves were used to form one biological replicate. In total we used nine and three biological replicates for metabolomics and proteomics, respectively. In order to build the network, we needed to have the same number of biological samples for metabolomics and proteomics. The drought treatment was applied at the vegetative stage of sugarcane as it has been identified to be critical of water demand (Ramesh, 2000); mainly because 70-80% of sugarcane yield is achieved during this developmental stage (Singh and Rao, 1987).

### Gas Exchange Measurements

Photosynthetic rate (*A*), transpiration rate (*E*) and stomatal conductance (*g*_s_) were measured using the portable photosynthesis system Li-6400 (LI-COR Biosciences, Inc.). Light curves were obtained with photosynthetic active radiation (PAR) values of 2000, 1500, 1000, 500, and 0 µmol m^-2^ s^-1^. The measurements were recorded between 8:00 to 11:00 am in the medium portion of leaf +1 completely expanded. Leaf temperature was kept at 25 °C during the IRGA analysis.

### Protein Extraction and Trypsin Digestion

Total protein was extracted from each biological sample, using the phenolic method according to Hurkman and Tanaka (1986), with minor modifications. Leaf tissues were ground into a fine powder in liquid nitrogen, and 200 mg was homogenized in 1 ml of extraction buffer (0.7 M sucrose, 0.5 M Tris-HCl, pH 7.5, 50 mM EDTA, 0.1 M KCl, 1% w/v polyvinylpolypirrolidone (PVPP), 2% v/v 2-mercaptoethanol, and 2 mM PMSF), by shaking for 30 min at 4°C. After, 800 µl of Tris-HCl saturated phenol pH 8.5 was added to the protein suspension and samples were shaken for 30 min at 4°C, the phases were separated by centrifugation (10,000 *g* for 30 min at 4°C). The supernatant was recovered and re-extracted with an equal volume of extraction buffer. This step was repeated two times. Proteins were precipitated by adding 5 vol of 0.1 M ammonium acetate in methanol and incubated overnight at 22°C. The samples were then centrifuged (9,000 *g*, 30 min at 4°C) and the resulting pellets were washed three times with 0.1 M ammonium acetate in methanol, followed by a wash with acetone. After complete evaporation of acetone at room temperature, the protein pellet was resuspended in 400 µl of solubilization buffer [7 M urea, 2 M thiourea, 0.4% v/v Triton X-100 and 10 mM dithiothreitol (DTT)]. Complete protein solubilization was achieved by vigorous shaking using a vortex for 2 min. Protein extracts were desalinized using Amicon Ultra-0.5 ml 3K-NMWL filter devices (Millipore Corporation). Total proteins were quantified using the Bradford method (Bradford, 1976). Fifty micrograms of proteins were denatured with 25 µl of 0.2% *Rapi*Gest (Waters, USA), reduced with 2.5 µl of 100 mM DTT and alkylated with 2.5 µl of 300 mM iodoacetamide. Trypsin digestion was performed with sequencing Grade Modified Trypsin (Promega) at a 1:100 (w/w) enzyme: protein ratio and proteins were incubated at 37°C overnight. After, 10 µl of 5% (v/v) trifluoroacetic acid (TFA) was added to the digested mixture to hydrolyze the *Rapi*Gest (Waters, USA). The peptide mixture was then desalted using ZipTip C18-columns (Millipore Corporation). The final volume of 40 µl was obtained by the addition of 20 mM ammonium formate (pH 10) solution containing 200 *f*mol/µl of rabbit phosphorylase (internal standard to data normalization and label free protein quantification- P00489) to the lyophilized, desalted peptide sample.

### LC-MS^E^ Analysis

The peptides mixture was analyzed by reverse-phase ultra-performance liquid chromatography (ACQUITY UPLC M-Class System with 2D Technology—Waters, USA) using a Synapt G2 HDMS (Waters, Manchester, UK). First dimension chromatographic separation was achieved using an AQUITY UPLC M-Class peptide BECH C18 columns (5 µm, 300 µm x 50 mm). Elution was performed using five different binary gradients with 20 mM pH 10 ammonium formate in acetonitrile, at a flow rate of 2 µl min^-1^. Eluted peptides from the first-dimension column were trapped in a Symmetry 2D C18 column (5 µm, 180 µm x 20 mm) and diluted, online, with acetonitrile containing 0.1% formic acid. Second dimension separation was performed in an AQUITY UPLC M-Class CSH C18 column (1.7 µm, 75 µm x 150 mm), using a binary gradient from 7% to 85% of acetonitrile with 0.1% formic acid, during 75 min, at a flow rate of 400 µl min^-1^.

Mass spectrometry acquisition was achieved in a Synapt G2 HDMS (multiplexed DIA—data-independent acquisition) (Waters, Manchester, UK) mass spectrometer equipped with ion mobility cell and a nanolockspray source in the positive ion and “V” mode.

The doubly-charged ion [(M + 2H)^2+^] was used for initial single-point and MS/MS fragment ions of GFP (Glu 1)-Fibrinopeptide B *m/z* 785,84,206 [(M + 2H)^2+^] (Waters, Corp., Milford, USA) were used as lock masses and instrument calibration, respectively. Data-independent scanning (MS^E^) experiments were performed by switching between low (3 eV) and elevated collision energies HDMS^E^ (19–45 eV), applied to the trap “T-wave” CID (collision-induced dissociation) cell filled with argon gas. Scan time of 0.8 s were used for low and high energy scans from *m/z* 50 to 2000 (Silva et al., 2006).

### Processing Parameters and Database Search

The raw data processing, protein identification and relative quantitative analyses were all performed using ProteinLynx Global Server v2.5.1 (PLGS, Waters). Proteins identifications were performed with PLGS searching into the SUCEST translated EST database (http://sucest-fun.org/), containing specific annotation of sugarcane ESTs (237.954 sequences) (Vettore et al., 2003). The processing parameters included: automatic tolerance for precursor and product ions, minimum of three fragment ions matched per peptide, minimum of seven fragment ions matched per protein, minimum of two peptides matched per protein, one possible trypsin missed cleavage, carbamidomethylation of cysteine as fixed modification and oxidation of methionine as variable modification, and a maximum false positive discovery rate (FDR) lower than 1%, determined based on the search of a reversed database, which was generated automatically using PLGS by reversing the sequence of each entry. To identify and quantify the proteins, the intensities of the spectra were calculated by the stoichiometric method, according to the internal standard, the sequence of rabbit phosphorylase (Uniprot entry: P00489), by MS^E^ analysis (Silva et al., 2006) and normalized using the PLGS auto normalization function. The amount and sequence of the matched peptides and protein *f*mols were obtained, based on the ratio of its three most abundant peptides (“High Top 3” method) determined in each individual experiment (Silva et al., 2006), considering the data from three biological replicates for each treatment. All protein hits were identified with a confidence of > 95%. Only those proteins identified in at least two out of three biological replicates were considered for analysis. Proteins identified but not quantified were removed from the analysis. The mass spectrometry proteomics data was deposited to the ProteomeXchange Consortium *via* the PRIDE (Vizcaíno et al., 2016) partner repository with the dataset identifier PXD014920 (username: reviewer11150@ebi.ac.uk; password: bs65Tv52)

### Functional Annotation and Classification

Gene Ontology (GO) annotation, to assign biological process terms, was performed based on Blast2GO software using the blastp algorithm whith the nr database (11/2018) and E-value ≤ 1e-05, the other parameters were setted as default (Conesa et al., 2005).

### Metabolites Extraction and Derivatization

Prior to metabolite extraction frozen leaf tissue (50 mg) was grounded under liquid nitrogen using a vibration mill (MM 301 Retch GmbH & Co, Haan, Germany) set to a frequency of 30 Hz s^-1^ for 45 s, with pre-chilled holders and 3 mm tungsten beads. Leaf metabolites were extracted by adding 500 µl of pre-chilled methanol (MeOH): chloroform (CHCl_3_): water (H_2_0) (6:2:2 v/v/v). The extract was vortexed vigorously, sonicated at 40 Hz s^-1^ for 15 min and centrifuged at 14.000 x *g* (Eppendorf Centrifuge 5415R) for 10 min at 4°C. The supernatant was filtered (Millipore filter PVDF 0.22 µm) and 100 µl of each sample was transferred to vials and evaporated until dryness.

Samples were derivatized according to Gullberg et al. (2004) with 30 µl of methoxyamine hydrochloride (15 mg ml^-1^) in pyridine for 16 h at room temperature. The samples were trimethylsilylated by adding 30 µl of N-methyl-N-(trimethylsilyl) trifluoroacetamide (MSTFA) containing 1% trimethylchlorosilane (TMCS), the resulting mixture stand at room temperature for 1 h. After silylation, 30 µl of heptane was added. Stable isotope reference compounds [1 mg ml^-1^ each of (^13^C_3_)-myristic acid, (^13^C_4_)-palmitic acid and (^2^H_4_)-succinic acid] were added in samples prior to derivatization and used as external standard for quality control. Derivatized samples were analyzed according to Gullberg et al. (2004). Blank control samples and a series of *n*-alkanes (C12–C40), which allowed retention indices to be calculated (Schauer et al., 2005) were also used.

### Analysis and Metabolites Identification by GC-TOF-MS

One microliter of each derivatized sample was injected splitless into a gas chromatograph 7890A (Agilent Technologies, Santa Clara, USA) coupled with a Comb-xt Autosampler (Leap Technologies, Carrboro, USA). A 20m x 0.18 mm i.d. x 0.18 µm film thickness, DB5 GC capillary column (Agilent Technologies) was used as the primary column and the secondary GC columns was a 0.69 m x 0.1 mm i.d. x 0.1 µm film thickness (Rxi-17 Restek, Bellefonte, USA). The injector temperature was 280°C, the septum purge flow rate was 20 ml min^-1^ and the purge was turned on after 60 s. The gas helium flow rate through the column was 1 ml min^-1^, the column temperature was held at 80°C for 2 min, then increased by 15°C min^-1^ to 305°C, and held there for 10 min. The column effluent was introduced into the ion source of a GC×GC/TOF-MS (Pegasus 4D, Leco Corp., St. Joseph, USA). The transfer line and the ion source temperatures were 280 and 250°C, respectively. Ions were generated by a 70-eV electron beam at an ionization current of 2.0 mA, and 10 spectra s^-1^ were recorded in the mass range *m/z* 45–800.

The ChromaTOF software v. 4.51 (Leco Corp., St. Joseph, USA) was used to perform baseline correction and export all MS files in NetCDF format. Peak detection, retention time alignment and library matching were performed using the TargetSearch package (Cuadros-Inostroza et al., 2009). Metabolites were identified by comparing their retention indexes (± - 2 s) and spectra (similarity > 600) against the compounds stored in the Golm-Metabolome-Database (http://csbdb.mpimp-golm.mpg.de/csbdb/gmd/gmd.html) (Kopka et al., 2005). Metabolites intensities were normalized by dry weight and total ion chromatogram (TIC).

### Statistical Analysis

Multivariate (Principal Component Analysis—PCA) univariate (ANOVA, FDR adjusted *p* ≤ 0.05) analysis and Heatmaps were done in MetaboAnalyst 4.0 (Xia et al., 2015). To reduce systematic variance and to improve the performance for downstream statistical analysis data were log-transformed and Pareto scaled prior to data analysis.

### Network Analysis

The network analysis was used to assess the relationship between metabolome and proteome data from PI *versus* 4DI treatment and PI *versus* 12DI, for both varieties. It was performed using the rCCA, which is a modification of the classical canonical correlation analysis (CCA), a multivariate statistical method used to assess correlations between two multivariate datasets (Gonzalez et al., 2008). This was performed using the mixOMICS v5.2 (Rohart et al., 2017) in R (http://www.R-project.org). Regularization parameters (λ_1_ and λ_2_) were estimated using the *tune.rcc* function, where a set of positive values chosen to evaluate the cross-validation score (*cv-score*) for each point in the network, achieving an optimal value for λ_1_ and λ_2_ that offered the highest *cv-score*. After, the *rcc* function was used to define the canonical correlations and the canonical variates between the two datasets. The unit representation plots were created using the *plotIndiv* function, in this plot each biological sample is projected into the XY-variate space. The correlation circle plots were created using the *plotVar* function, where all variables are represented through their projections onto the planes, highlighting the direction and correlation values of each variable. The *network* function was used to produce relevant networks from the similarity matrix derived from rCCA. The threshold was set to 0.99 and only the associations exceeding this threshold were shown. This threshold values were stringent to obtain biologically interpretable networks (Gonzalez et al., 2012). In order to analyze the created networks they were exported to Cytoscape 3.3 network visualization software (Shannon et al., 2003). The used layout was the organic, with the diameter of the node relative to the number of edges and number of connected edges. The correlation values is highlighted by the intensity of edge color ranging from dark green (negative correlation) to dark red (positive correlation).

## Results

### Changes in Photosynthetic Capacity Under Drought

**Figure 1:**
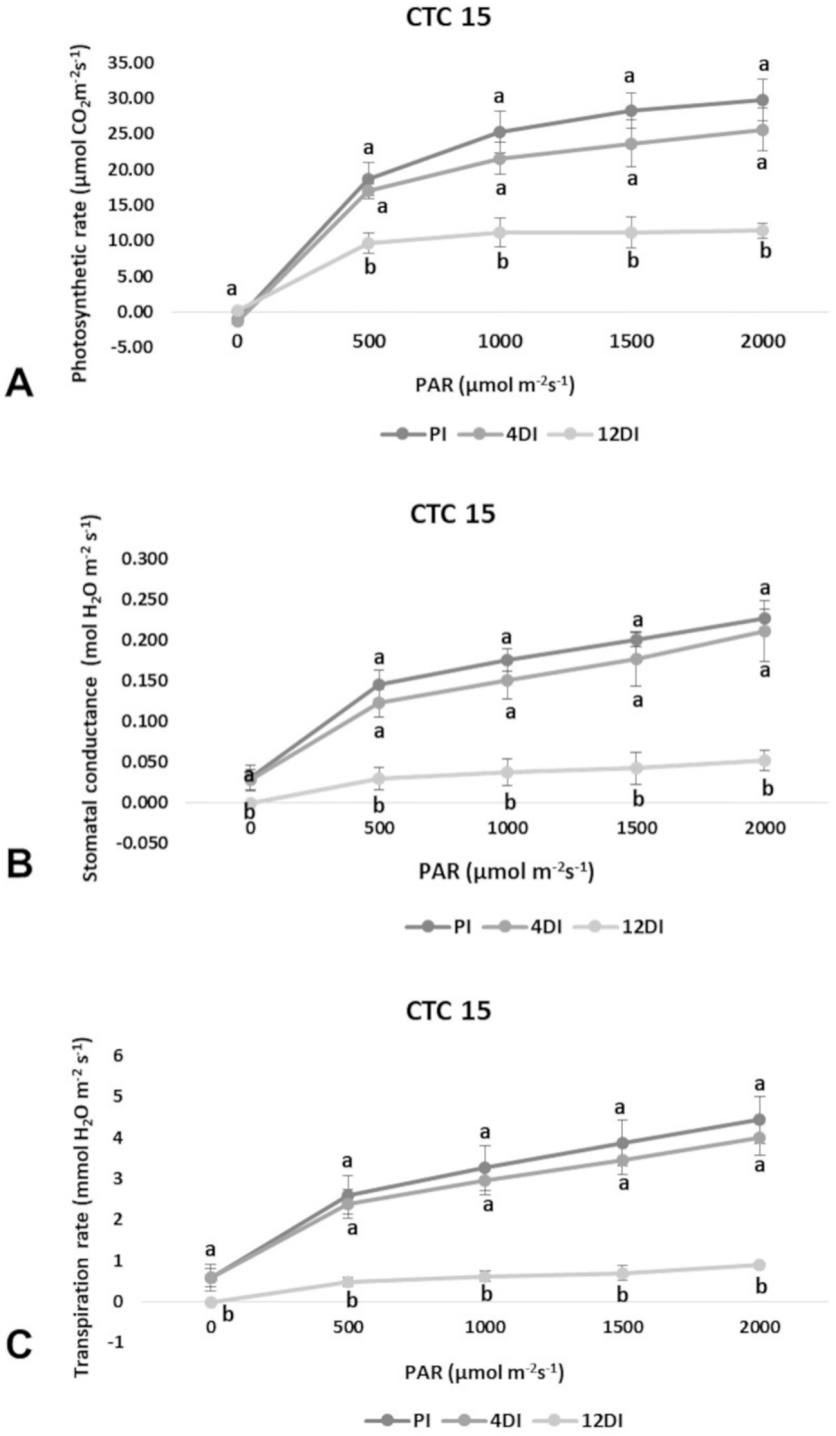
Effects of drought on photosynthetic rate—*A* **(A)**, stomatal conductance—*g*s **(B)**, and Transpiration rate—*E* **(C)** in the variety CTC15 (drought-tolerant). Comparison among permanently irrigated (PI), 4 days without irrigation (4DI) and 12 days without irrigation (12DI). Letters indicate differences significant by Tukey’s test at 5% probability level.

**Figure 2:**
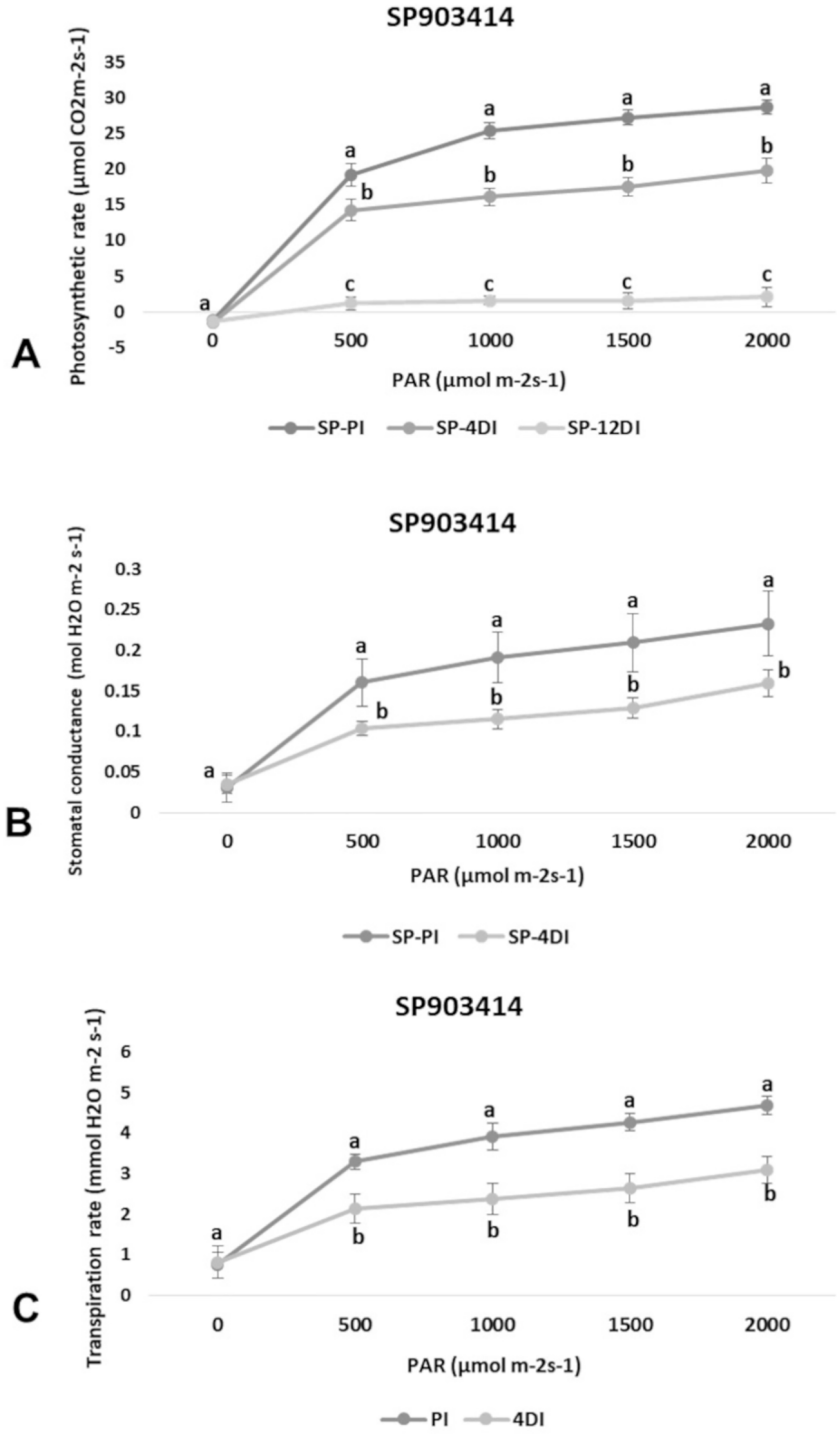
Effects of drought on photosynthetic rate—*A* **(A)**, stomatal conductance—*g*s **(B)**, and Transpiration rate—*E* **(C)** in the variety SP90-3414 (drought-susceptible) cultivar. Comparison between permanently irrigated (PI) and 4 days without irrigation (4DI) plants. Letters indicate differences significant by Tukey’s test at 5% probability level.

Photosynthetic rate (*A*), transpiration (*E*) and stomatal conductance (*g*_s_) were determined in leaves (+1) to verify the physiological effects of drought upon each variety, under 4DI and 12DI, corresponding to 40% and 25% FC, respectively. As expected, it was possible to observe differences in performance between tolerant and susceptible varieties, under drought. CTC15 showed no major changes in gas exchange at the initial drought exposure, with no significant reduction in photosynthesis, transpiration and stomatal conductance between PI and 4DI (**Figure 1**). On the other hand, photosynthesis diminished at 12DI by around one third of the values observed in PI. These results indicated the ability of the tolerant variety to maintain key physiological process, during a short-term drought. In SP90-3414, photosynthesis reduced significantly at 4DI and mainly in 12DI plants (**Figure 2**). Leaf transpiration and stomatal conductance also diminished after 4DI, compared to the PI (**Figure 2**). In comparison to the tolerant variety, SP90-3414 was already suffering a limitation in the photosynthetic capacity at 4DI and was mainly limited at 12DI, probably due to a prolonged stomatal closure, leading to a reduced carbon fixation. During the drought treatment, SP90-3414 variety showed signs of stress, mainly at 12DI such as wilting leaves and progressive leaf rolling.

### Proteome Changes in Sugarcane Leaves in Response to Drought

**Figure 3:**
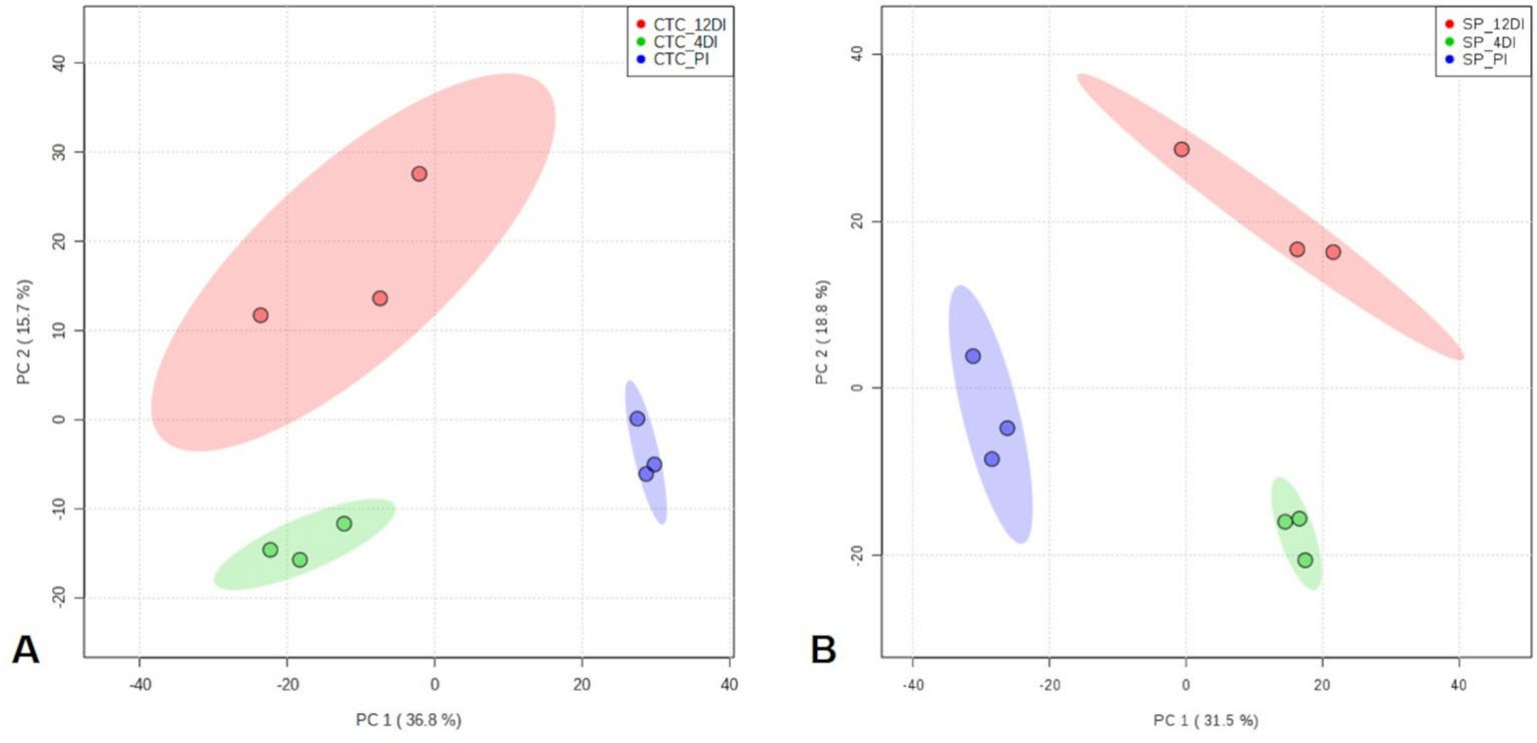
Principal Component Analysis (PCA) score plots of proteins identified in the three treatments (permanently irrigated (PI), 4 days without irrigation (4DI) and 12 days without irrigation (12DI) in sugarcane leaves. **(A)** Drought-tolerant variety CTC15. **(B)** Drought-susceptible variety SP90-3414.

**Figure 4:**
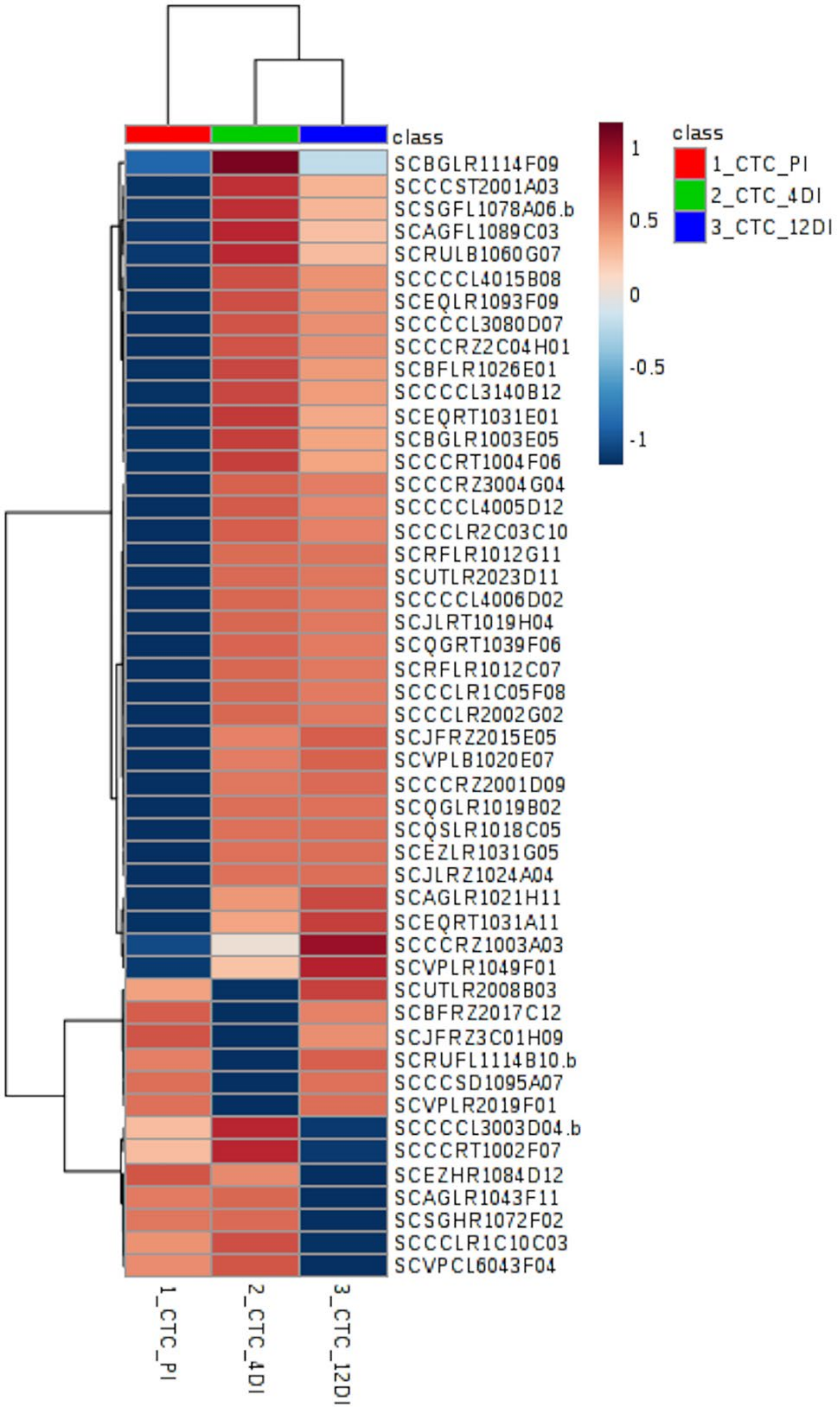
Heatmap based on differentially abundant proteins (*p* ≤ 0.05, FDR adjusted) from the drought-tolerant variety CTC15. PI (permanently irrigated), 4DI 4 days without irrigation and 12DI 12 days without irrigation.

By using spectral counting-based label free quantification and searching the SUCEST project database (http://sucest-fun.org/), in total, 1650 and 1750 proteins were identified and quantified at 95% confidence level, in CTC15 and SP90-3414 varieties, respectively. Venn diagram was performed to identify the common and unique proteins in each variety (**Supplementary Figure 1**), providing an overview of whether the changes represented common or specific responses. In CTC15 and SP90-3414 plants were found 1218 and 1336 common proteins, respectively. In both varieties, 12DI plants showed the highest number of unique proteins (200—CTC15; 182—SP90-3414) followed by 4DI (176—CTC15; 142—SP90-3414) and PI (56—PI; 90—SP90-3414) plants. The unique proteins (**Supplementary Table 1**) were removed from the matrix submitted to statistical analysis. Proteomics changes in response to drought were first examined to identify the protein distribution within the treatments of each variety. To reduce the dimensionality of the data and visualize samples grouping tendency and outliers, a PCA was performed (**Figures 3A, B**). All data were lying inside the 95% confidence region (Hotelling’s T^2^ ellipse). The score plot (**Figures 4A, B**) showed a clear trend of treatments separation, indicating a metabolic adjustment to drought at protein level, in both varieties. The first principal component (PC1) separates the PI plants, of each variety, from the 4DI and 12DI plants explaining 36.8% (**Figure 3A**) and 31.5% (**Figure 3B**) of the total variation, respectively. 4DI and 12DI plants were separated by PC2, representing 15.7% (**Figure 3A**) and 18.8% (**Figure 3B**) of the total variance in CTC15 and SP90-3414 varieties, respectively. To identify if both varieties would present similar responses to water deficit, two Venn diagrams were constructed, based on the differentially abundant (*p* ≤ 0.05) and unique proteins (**Supplementary Figure 2**). Considering the differentially abundant ones, only two proteins (1.9%) [fumarylacetoacetate hydrolase domain-containing protein 1 (SCJFRZ2015E05) and prli-interacting factor l-like (SCRULB1060G07)] were common between CTC15 and SP90-3414 varieties. However, 100 unique proteins (13.4%) were common to both varieties (**Supplementary Figure 3**), most of them belongs to the category *Metabolic process*—GO:0008152 (25%) and *Cellular process*—GO:0009987 (17%).

### Protein Changes in CTC15 and SP90-3414 in Response to Drought

By performing Anova (FDR adjusted *p* ≤ 0.05) 49 differentially abundant proteins were identified in the tolerant variety (**Supplementary Table 2**), their distribution can be observed in the heatmap (**Figure 4**). Aiming to identify which biological processes were most strongly related to drought stress, these proteins were classified, using Blast2GO. The five most represented process were: *Metabolic process*—GO:0008152 (30%), *Translation*—GO:0006412 (26%), *Cellular process*—GO:0009987 (20%), *Catabolic process*—GO:0009056 (20%), and *Biosynthetic process*—GO:0009058 (12%). From the 49 differentially abundant proteins (**Supplementary Table 2**), 35 (71%) increased in abundance upon drought, while 6 and 7 proteins had its content reduced upon drought at 4DI and 12DI, respectively. For instance, at 4DI a zeaxantin epoxidase (SCBFRZ2017C12), a polygalacturonanse precursor (SCJFRZ3C01H09), ndph-dependent reductase (SCRUFL1114B10.b), and two ribosomal proteins, had their abundance significantly reduced. At 12DI we observed the reduction in content of the following proteins: glycine cleavage h-protein (SCEZHR1084D12), plasma membrane atpase (SCCCRT1002F07), ribosomal protein (SCAGLR1043F11), elongation factor (SCCCCL3003D04.b), and a fumarylacetoacetate hydrolase domain (SCVPCL6043F04). Most of these highly abundant proteins at 4DI and 12 DI were related to *Translation process* (GO:0006412). Some of the proteins found are known to be drought responsive (i.e. two glutathione-s-transferase (SCBGLR1003E05 and SCAGFL1089C03), ferredoxin-sulfite reductase (SCQGLR1019B02), lipoxygenase (SCJLRZ1024A04), nadh-ubiquinone oxidoreductase (SCAGLR1021H11), vacuolar atpase b subunit (SCQGRT1039F06), endoplasmin (SCSGFL1078A06.b), 60s ribossomal protein (SCRFLR1012C07, SCBFLR1026E01, SCCCCL3140B12).

**Figure 5:**
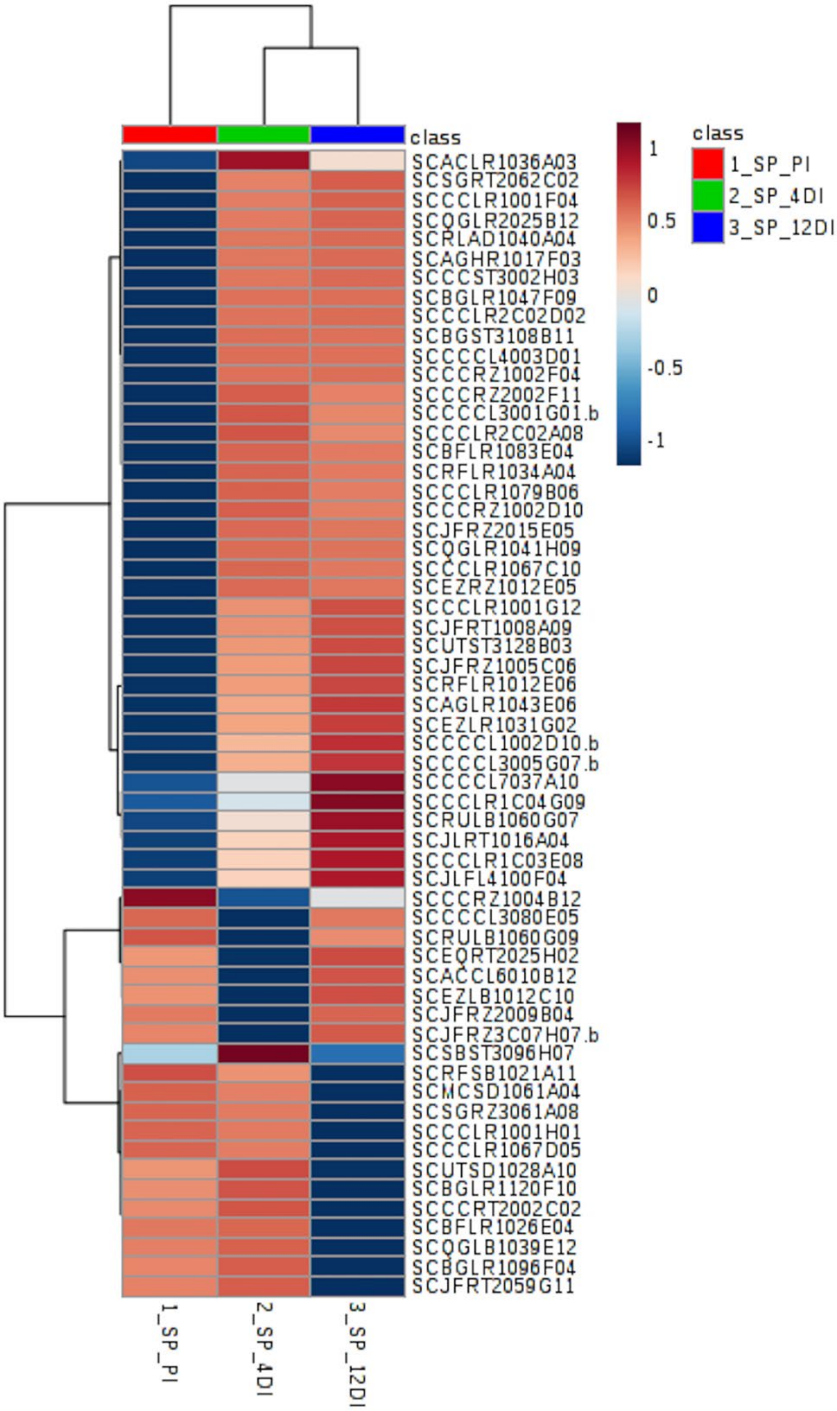
Heatmap based on differentially abundant proteins (*p* ≤ 0.05, FDR adjusted) from the drought-susceptible variety SP90-3414. PI (permanently irrigated), 4DI 4 days without irrigation and 12DI 12 days without irrigation.

In SP90-3414 plants, 59 proteins were differentially abundant (FDR adjusted *p* ≤ 0.05) (**Supplementary Table 3**). Their distribution pattern can be observed by the heatmap in **Figure 5**. The five most representative biological process found for these proteins were: *Metabolic process*—GO:0008152 (29%), *Cellular process*—GO:0009987 (22%), *Generation of precursor metabolites and energy*—GO:0006091 (10%), *Carbohydrate metabolic process*—GO:0005975 (10%), and *Response to stress*—GO:0006950 (8%) (**Supplementary Table 3**). From the 59 proteins, 15 (25.4%) were differentially abundant in all treatments and most of them were related to the *Metabolic process* (GO:0008152) category. From these proteins, 60%, 27%, and 13% were highly abundant at 12DI, 4DI, and PI, respectively. Some of the proteins found were abscisic acid stress ripening protein (SCCCST3002H03), glutathione s-transferase (SCJFRT1008A09), glutaredoxin (SCCCLR1C03E08), 60s ribosomal protein (SCBGLR1120F10), light-induced protein (SCCCRT2002C02), and mRNA binding precursor (SCRFSB1021A11). As observed for CTC15, most of the proteins (52.5%) accumulated at 4DI and 12DI, in comparison to PI. Glutathione reductase (SCRLAD1040A04), ferredoxin-chloroplastic-like (SCJLFL4100F04), glutathione transferase (SCCCCL4003D01), heat shock (SCCCCL3005G07.b), and phospholipase (SCAGLR1043E06) are some of the proteins found. Eight proteins (13.5%) were highly abundant at PI and 4DI, in comparison to 12DI and five proteins (8.4%) were highly abundant at PI and 12DI (**Supplementary Table 3**). In this variety, photosynthesis related proteins such as thylakoid membrane phosphoprotein (SCMCSD1061A04), chlorophyll a-b binding protein (SCEZLR1031G02), photosystem i reaction center subunit (SCQGLR2025B12) were found.

### GC-TOF-MS Based Metabolic Changes in Sugarcane Leaves in Response Drought

**Figure 6:**
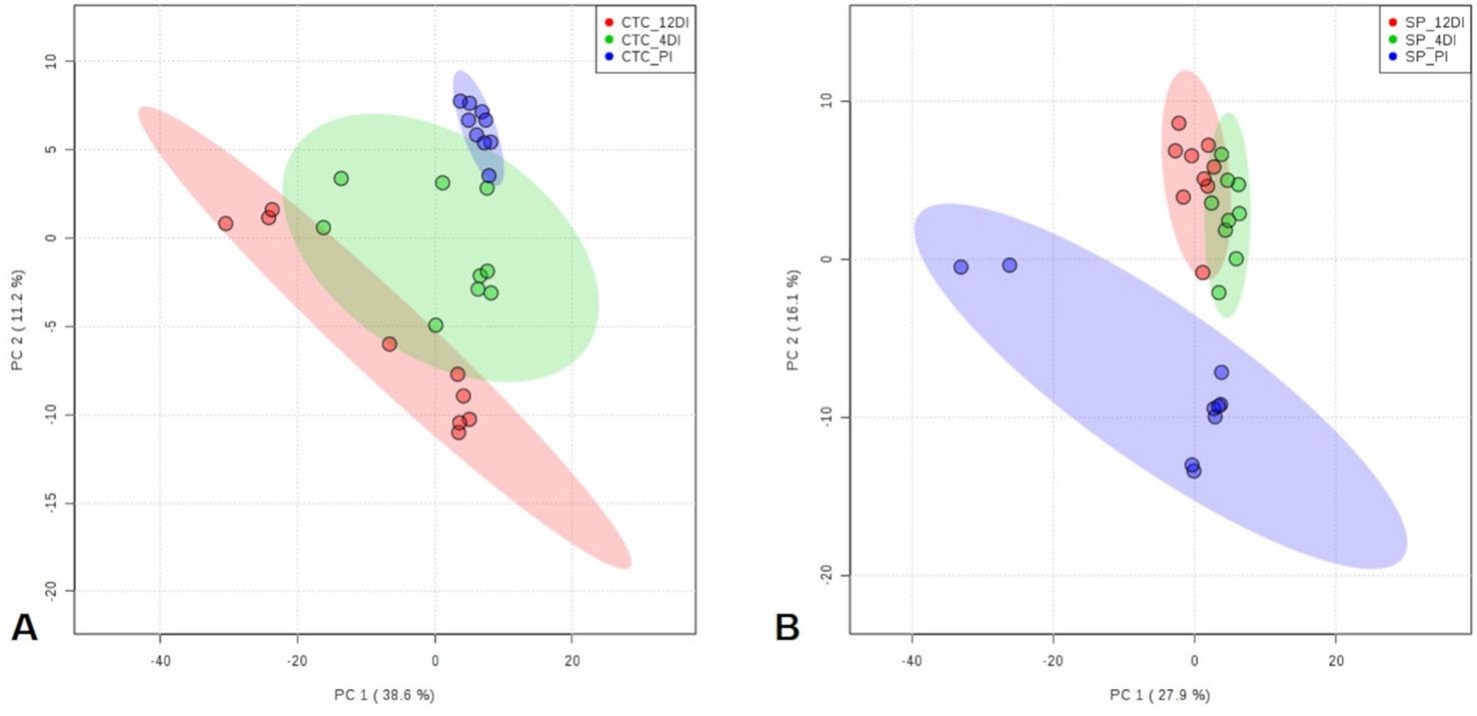
Principal Component Analysis (PCA) score plots of metabolites identified in the three treatments permanently irrigated (PI), 4 days without irrigation (4DI) and 12 days without irrigation (12DI) in the leaves. **(A)** Drought-tolerant variety CTC15. **(B)** Drought-susceptible variety SP90-3414.

**Figure 7:**
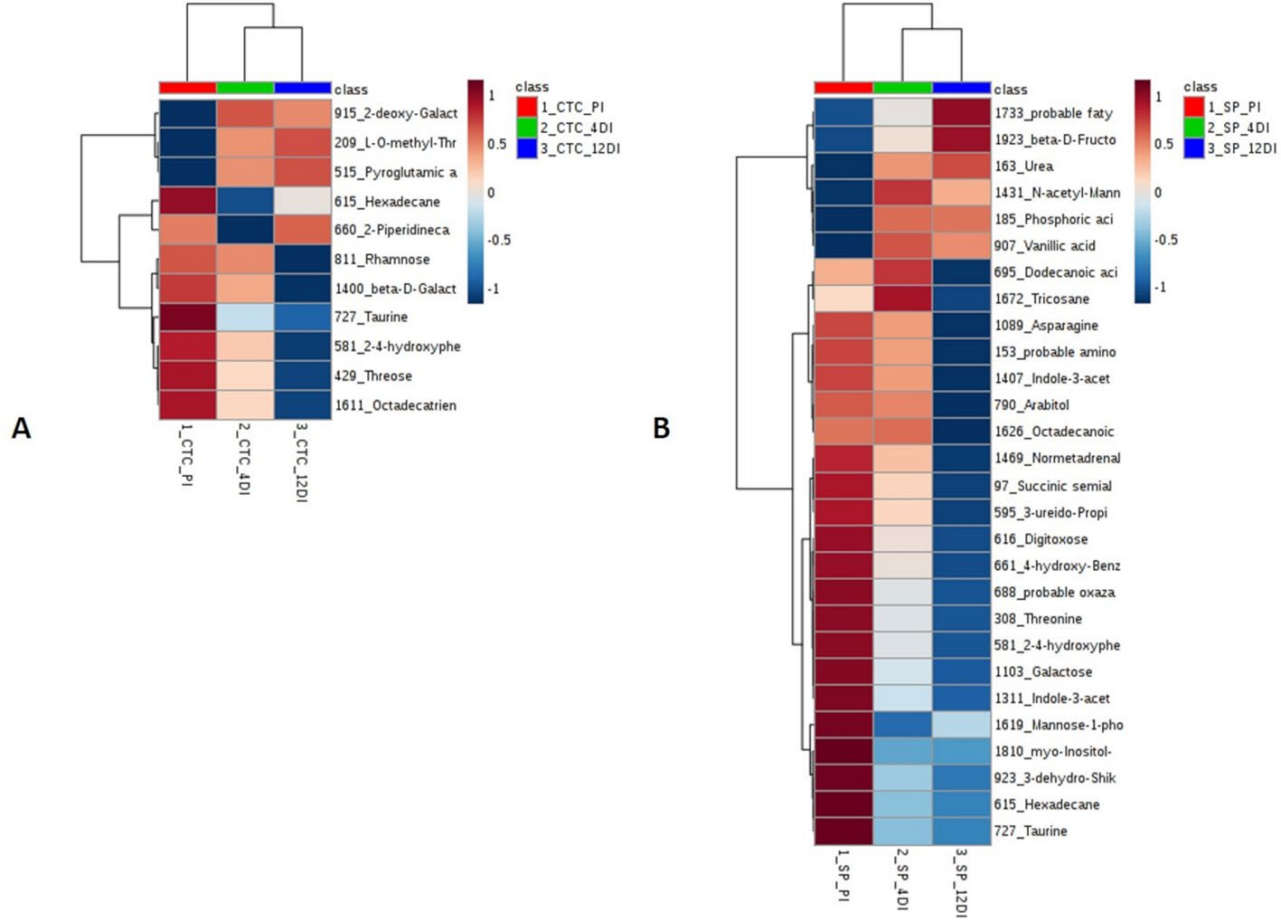
Heatmap based on differentially abundant metabolites (*p* ≤ 0.05, FDR adjusted). **(A)** drought-tolerant variety CTC15 and **(B)** drought-susceptible variety SP90-3414. PI (permanently irrigated), 4DI 4 days without irrigation and 12DI 12 days without irrigation.

Since metabolic regulation is a major mechanism to overcome drought in plants, metabolomics analysis was performed aiming to obtain an overview about the metabolic changes occurring in tolerant and susceptible sugarcane varieties, exposed to drought. After manual inspection, we could annotate a total of 104 and 97 metabolites, for CTC15 and SP90-3414, respectively (**Supplementary Tables 4** and **5**). Few metabolites from CTC15 (probable a Glycosyl and Amino acid derivative) and SP90-3414 (probable Amino acid derivative, probable Fatty Acyls, probable Carboxylic acid, Probable Steroid/steroid derivative and probable Oxazanine) were annotated based on the best mass spectral match from the database, however, their identity refered to metabolites that were, probably, not previously reported in plants. They were kept in our analysis and annotated based on their chemical category. Organic acids, sugars, lipids and others were the main classes found in both varieties (**Supplementary Tables 4** and **5**). Venn diagram was performed to identify the common and unique metabolites in each variety (**Supplementary Figures 4A, B**), providing an overview of whether the changes represented common or specific responses. In CTC15 and SP90-3414 plants were found 97 and 87 common metabolites, in at least two treatments, respectively (**Supplementary Figures 4A, B**). The number of unique metabolites found were 2 (PI), 1 (4DI), and 4 (12DI), in CTC15 and 5 (PI), 3 (4DI), and 2 (12DI) in SP90-3414 (**Supplementary Figures 4A, B**). To obtain the metabolite distribution pattern within the treatments, of each variety, the unique metabolites (found in a single treatment) were removed from the matrix submitted to statistical analysis. To investigate how each variety responded to drought, a PCA was performed for each (**Figures 6A, B**). The PCA score plot revealed differences in the metabolome of each treatment, suggesting a metabolic reconfiguration in response to drought. All data were lying inside the 95% confidence region (Hotelling’s T^2^ ellipse). The sum of the first two principal components explained 49.8% and 44% of the variation found in CTC15 and SP90-3414 varieties, respectively. In CTC15, despite the dispersion inside PI and 4DI samples, components 1 and 2 separated PI from 4DI and 12DI (**Figure 6A**). The same dispersion was observed for SP90-3414-PI samples. For this variety, component 2 has a tendency to separate PI from samples submitted to drought 12DI (**Figure 6B**). To identify the metabolites significantly affected by drought, one-way Anova (FDR adjusted *p* ≤ 0.05) was performed for each variety and revealed 10 and 28 metabolites differentially abundant in CTC15 and SP90-3414, respectively (**Supplementary Tables 3** and **4**). Sugars, organic acids and lipids were the main classes of metabolites identified. Prior to Anova, samples, from both varieties, showing high variation were removed. The distribution of differentially abundant metabolites, in each variety, was represented by heatmaps (**Figure 7**). Three differentially abundant metabolites [2-(4-hydroxyphenyl)-Ethanol], hexadecane and taurine were found in both varieties. 2-(4-hydroxyphenyl)-Ethanol is a phenolic compound, reduced in CTC15 at 12DI, in comparison to PI and 4DI. In SP90-3414, it accumulated at PI. Hexadecane, an alkane, accumulated at PI in both varieties. Taurine, and amino acid reduced under drought. In both varieties, most of the differentially metabolites accumulated in PI and reduced in response to drought. In the CTC15 variety, the metabolites O-methyl-L-Threonine, 2-piperidinecarboxylic acid, and pyroglutamic acid were indicated as differentially abudant because they were not found at 12DI, however, any of them changed between 4DI and 12DI and will not be discussed.

### Acessing Metabolite-Protein Networks in Sugarcane Leaves Under Drought by rCCA

**Figure 8:**
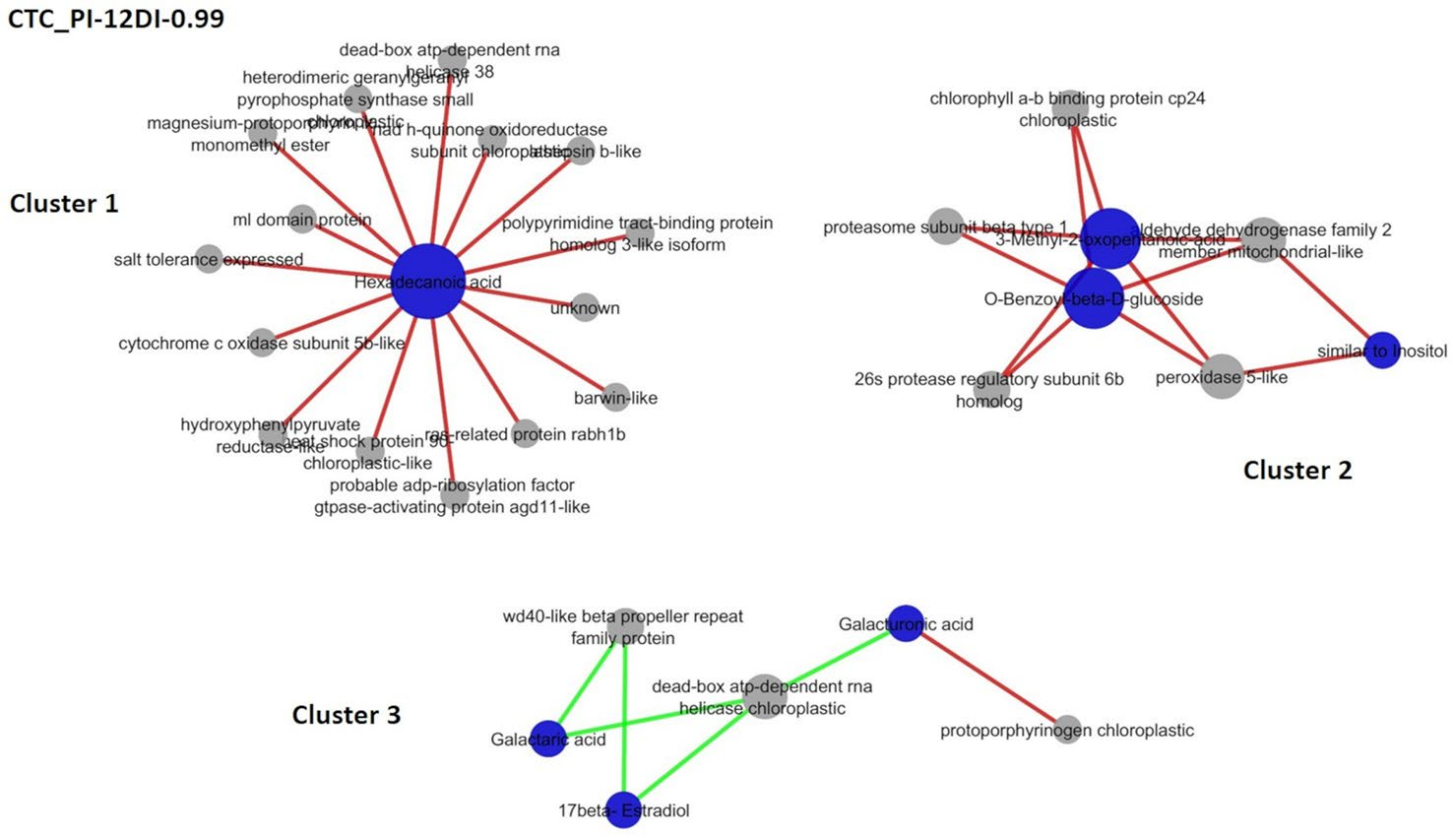
Correlation network depicting correlations derived from rCCA between metabolites and proteins from drought-tolerant variety CTC15. CTC-PI-12DI networks. PI (permanently irrigated) and 12DI (12 days without irrigation 12DI).

**Figure 9:**
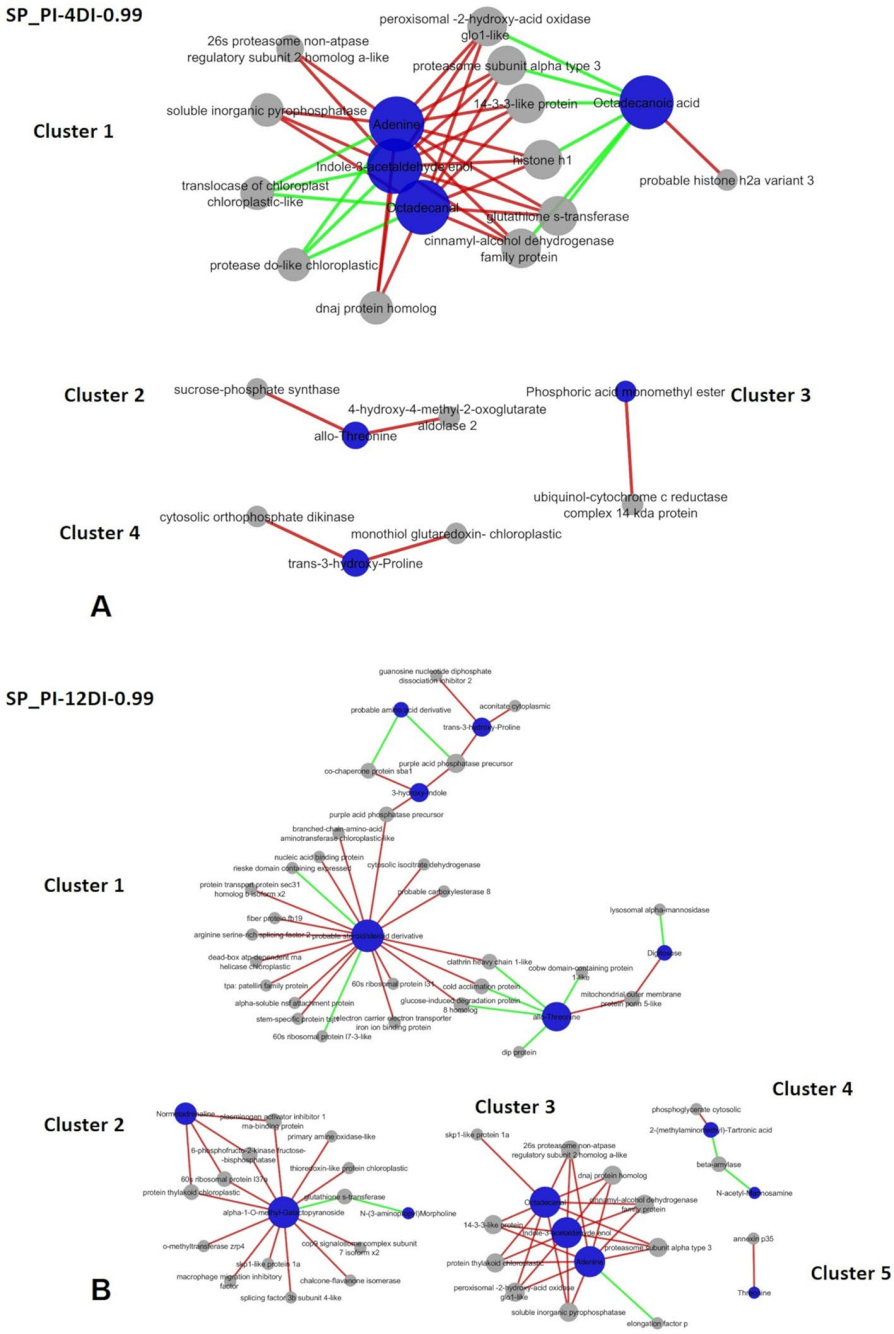
Correlation network depicting correlations derived from rCCA between metabolites and proteins from drought-susceptible variety SP90-3414. **(A)** SP-PI-4DI networks. **(B)** SP-PI-12DI networks. PI (permanently irrigated, 4DI (4 days without irrigation) and 12DI (12 days without irrigation 12DI).

It is expected that under stress conditions plants adjust their metabolic responses, in order to maintain its physiological homeostasis. Aiming to access part of these responses, rCCA was performed and metabolite–protein networks were identified, at a highly stringent threshold of 0.99 (**Figures 8** and **9**). Due to the high stringency of the analysis.

Networks were performed for each variety separately, combining PI-4DI and PI-12DI data, aiming to access the metabolites and proteins correlations at each level of stress. Given the linear correlation of variables used in rCCA, a correlation in the same direction is called positive and in the opposite direction is called a negative correlation. The number of positive and negative correlations and the complete description of each network are reported in the **Supplementary Tables 6** and **7**. The unit representation and the correlation circle plot of the rCCA can be visualized in the **Supplementary Figures 5** and **6**, respectively. The unit representation plot of the tolerant variety in PI-4DI analysis (**Supplementary Figure 5A**) did not show a clear separation between PI and 4DI. This might be due the fact that this tolerant variety, after 4 days of drought, shows no major differences in relation to control plants. In this sense, the network correlation is not represented. **Supplementary Figures 5B–D** show a clear separation between treatments of tolerant and susceptible varieties, CTC PI-12DI, SP PI-12DI, and SP PI-4DI, respectively. As shown in **Supplementary Figure 5A**, the plot did not revealed differences between CTC-15-PI and CTC-15-4DI. For this reason, the network correlation analysis was not done for CTC15-PI-4DI. All correlation circle plots (**Supplementary Figure 6**) showed a large number of metabolites and proteins with a high correlation value. Due to the large number of variables in our datasets, a clearer view of these correlations can be highlighted in the correlation networks. In the tolerant variety, three clusters were found in PI-12DI (lambda 1 = 1, lambda 2 = 0.99, cv-score = 0.97). In terms of biological process *Metabolic process* (GO:0008152) and *Response to stress* (GO:0006950) represented most of the proteins found in the CTC15-PI-12DI network (**Supplementary Table 6**). CTC15-PI-12DI cluster 1 (**Figure 8**) is the biggest one, refers to Hexadecanoic acid (ID 1391, positively correlated to 15 proteins). Cluster 2 refers to three metabolites: 3-Methyl-2-oxopentanoic-acid (114), O-Benzoyl-beta-D-glucoside (1847), and similar to Inositol (1350), positively correlated to five proteins. Cluster 3, the smaller one, contains three metabolites: Galacturonic acid (1268), Galactaric acid (1377), and 17-beta-Estradiol (1955), correlated to three proteins. In this cluster, the only positive correlation was between Galacturonic acid and protoporphyrinogen chloroplastic (SCMCRZ3064A10). The enzymatic role of the proteins identified in the networks were accessed and grouped according to metabolic pathways (**Supplementary Table 6**), based on EC number.

In SP90-3414 variety, four and five clusters were found for PI-4DI (lambda 1 = 1e-07, lambda 2 = 0.049, cv-score = 0.93) and PI-12DI (lambda 1 = 0.018, lambda 2 = 1e-04, cv-score = 0.99), respectively. Based on the biological process *Cellular* (GO:0009987) and *Metabolic process* (GO:0008152) were the most represented in SP90-3414 PI-4DI and PI-12DI networks (**Supplementary Table 7**; **Figures 9A, B**). PI-4DI cluster 1 is the biggest, containing four metabolites (Adenine—ID 1179, Indole-3-acetaldehyde enol—ID 1311, Octadecanal—ID 1359, Octadecanoic acid—ID 1626) and 12 proteins. Octadecanoic acid was negatively correlated with six proteins that were positively correlated with the other three metabolites. Cluster 2 consist of allo-Threonine (ID 297), positively correlated to two proteins. Cluster 3 is based on Phosphoric acid monomethyl ester (ID 108), positively correlated to one protein and, cluster 4 is based on trans-3-hydroxy-Proline (417), positively correlated with two proteins. In SP90-3414 PI-12 five clusters were found. Cluster 1, the biggest, is composed of six metabolites (Norvaline—ID 153, allo-Threonine—ID 297, trans-3-hydroxy-Proline—id 417, Digitoxose—ID 616, 3-hydroxy-Indole—ID 793, Lithocholic acid—ID 2412) correlated (positive and negative) with 27 proteins. Cluster 2 is based on three metabolites (N-(3-aminopropyl) Morpholine – ID 688, alpha-1-O-methyl-Galactopyranoside—ID 981, Normetadrenaline—1469) correlated (positive and negative) with 13 proteins. Cluster 3 is based on three metabolites (Adenine—1179, Indole-3-acetaldehyde enol—ID 1311, Octadecanal—ID 1359) correlated with 10 proteins. One negative correlation was found in this cluster (adenine correlation with one protein). The metabolites in cluster 3 are the same found in SP90-3414 PI-4DI cluster 1. Cluster 4 is based on two metabolites [2-(methylaminomethyl)-Tartronic acid—ID 453, N-acetyl-Mannosamine—ID 1431] correlated (positive and negative) with two proteins. Cluster 5 is based on Threonine (308) positively correlated with a single protein. Regarding the enzymatic role of the proteins found in SP90-3414 networks, a completely different set of elements were observed. The common elements are mostly related to photosynthesis and oxidation, reflecting the major impacts caused by drought. In PI-4DI, 3 elements were unique in this condition and 15 were common to 12DI. This suggests that photosynthesis and oxidation have a higher impact at 4DI. However, at 12DI most of the elements were unique to this condition. These elements belong to important primary and secondary metabolic pathways, indicating that at 12DI, the susceptible variety could be requiring a different metabolic adjustment, to face drought. The comparison between varieties, at the same condition, revealed clear differences in response to drought (**Supplementary Table 7**). At 4DI, while CTC15 required amino acid and photosynthetic ajdustments while SP90-3414 needed to adjust metabolism mainly in reponse to oxidative stress. Twelve elements were common to both varieties, other twelve were found only in CTC15 and a lower number, six were found in SP90-3414. This could also indicate that both varieties start to perceived drought at 4DI. The difference is that, CTC15 sucssesfully adjust its metabolism in order to keep stomatic conductance and photosynthesis, being able to maintain homestasis. On the other hand, to maintain cellular homeostasis, SP90-3414 needs to adjust its metabolic and physiological responses by closing stomatal conductance and reducing photosynthesis, already at 4DI. The changes between both varieties, at 12DI, showed that metabolic activity is still ongoing but, different pathways were required. CTC15 is dealing with amino acid and lipids degradation and oxidation process, while SP90-3414 faces alterations in carbon fixation and eletron transport chain. These results indicated that each variety demands a specific adjustment to metabolism maintanence and survival, under drought.

## Discussion

Network biology is a rapidly developing area of research, which states that biological processes are not chiefly controlled by individual molecules or by discrete, unconnected linear pathways (Charitou et al., 2016). Here, we propose the application of rCCA to explore correlation structures between metabolites and proteins, as an exploratory strategy to improve our knowledge about leaf metabolism in sugarcane under drought, which is one of the main factors negatively impacting sugarcane productivity worldwide.

### Physiological Responses of CTC15 and SP90-3414 to Drought

Plants have developed physiological mechanisms to save water during drought periods. As an example, stomatal closure is the first line of defense against drought (Silva et al., 2013). Therefore, photosynthesis is particularly impacted to drought once stomata closure reduces CO_2_ diffusion to the fixation sites, in leaf mesophylls (Ghannoum, 2009). In accordance to this, all parameters measured (*A*, *E* and *g* *_s_*) were reduced at 4DI and 12DI in SP90-3414 (**Figure 2**). For the susceptible variety, stomata closure occurs earlier than observed in the tolerant, in order to reduce water loss and maintain plant metabolism. The reduction in photosynthetic capacity was already reported for several sugarcane varieties susceptible to drought (Rodrigues et al., 2009; Rodrigues et al., 2011; Kido et al., 2012; Silva et al., 2013), supporting our observations with SP90-3414. For the tolerant variety, the observed differences were not statistically significant between PI and 4DI (**Figure 1**), only at 12DI. Similar observations were reported for the tolerant sugarcane variety SP83-5073. While analyzing *A*, *E,* and *g* *_s_* the authors found that tolerant plants behaved exactly as the control plants under 4 days of drought.

### Proteome Dynamics of Sugarcane Leaves Under Drought

It is known that in C_4_ plants photosynthetic inhibition under drought is mainly due to stomatal closure (Ghannoum, 2009), resulting in lower carbon fixation, followed by the over-reduction of the electron transport system and production of ROS (Nouri et al., 2015). Although physiological data showed that tolerant variety, at 4DI (**Figure 1**), did not differ significantly from control plants, the proteome exhibited differences, indicating that protein changes may precede physiological responses to drought, in this variety. Besides, no protein directly involved in carbon fixation was differentially abundant in CTC15 (**Supplementary Table 2**), while, in SP90-3414 four proteins [chlorophyll a-b binding protein (SCEZLR1031G02), photosystem I reaction center subunit (SCQGLR2025B12), ferredoxin- chloroplastic-like (SCJLFL4100F04), ribulose-phosphate 3-epimerase (SCAGHR1017F03)] accumulated under drought (**Supplementary Table 3**). The proteomic adjustment of each variety to drought seems to require different strategies. Considering the GO terms, most of the differentially abundant proteins from CTC15 and SP90-3414 belong to *Metabolic process* (GO:0008152). Considering that this term is a general one, attributed to a variety of proteins, this observation was not a surprise. Instead, the most represented terms, such as *Translation* (GO:0006412) in CTC15, *Generation of precursor metabolites and energy* (GO:000609) and *Response to stress* (GO:0006950), found in SP90-3414, contributed to reveal variety differences in response to drought.

In general, elevated levels of proteins involved in translation can be beneficial for maintenance of protein synthesis under drought (Merewitz et al., 2011). Accordingly, different proteoforms of them were found in our work. 40s (SCCCCL3080D07), 60s (SCRFLR1012C07, SCBFLR1026E01, SCCCCL3140B12), ribosomal s8 (SCEQRT1031E01), and 26s proteasome regulatory subunit (SCVPLR1049F01, SCCCCL4005D12) proteins accumulated under drought. On the other hand, two ribosomal proteins [30s (SCCCSD1095A07), 60s (SCVPLR2019F01)] accumulated at 12DI and PI, and two proteins (eukaryotic translation initiation factor 3, ribosomal protein rps20) accumulated at 4DI and PI. Elongation factor is a core translational protein that catalyzes the initiation and elongation of newly growing peptide chains (Wan and Liu, 2008). Two proteoforms of elongation factors were found, SCVPLB1020E07 accumulating under drought and SCCCCL3003D04.b accumulating at PI and 4DI. This differential change may indicate that the form SCVPLB1020E07 is directly involved maintaining the synthesis of proteins directly involved with drought. It is important to highlight that four proteins, differentially abundant, related to translation [60s (SCBGLR1120F10) and 40s (SCCCLR1001H01, SCCCCL3080E05, SCCCLR2C02A08)] were found in SP90-3414. As mentioned above, the terms *Response to stress* (GO:0006950) and *Generation of precursor metabolites and energy* (GO:000609) contribute to characterize the protein adjustment of SP90-3414 variety, to drought. Among the *Response to stress* (GO:0006950), one dehydrin (dhn2-like protein—SCCCLR1001F04) was found, accumulating during drought. Dehydrins are a group of late embryogenesis abundant (LEA) proteins, which protects cellular membranes and organelles during dehydration (Wahid and Close, 2007) and it is known to be induced by drought (Wahid and Close, 2007; Hand et al., 2011; Ferreira et al., 2017). This protein was differentially abundant only in the susceptible variety, suggesting that membrane protection is earlier required in SP90-3414 than in tolerant variety. Heat shock protein that belongs to response to stress is another common protein responsive to drought (Wang et al., 2003). In our work, one heat shock protein was found in each variety. In CTC15, the abundance of SCCCRZ1003A03 increased according to drought duration. In SP90-3414, SCCCCL3005G07.b accumulated in response to drought. The *Generation of precursor metabolites and energy* (GO:000609) category include proteins related to the primary metabolism, such as carbon fixation, glycolysis, citric acid cycle (TCA), and electron transport chain. These proteins were found in SP90-3414, accumulating in response to drought. Since primary metabolism can be generally defined as essential for survival (Aharoni and Galili, 2011), our results suggest that the susceptible variety priorize the metabolism of energy generation as a strategy to face drought. Two differentially abundant proteins, prli-interacting factor (SCRULB1060G0) and fumarylacetoacetate hydrolase domain (SCJFRZ2015E05), were differentially abundant in both varieties. In CTC15 it accumulated in response to drought while, in SP90-3414, was gradually accumulating in response to drought. This protein is related to ubiquitin-dependent protein catabolic process and was found in sugarcane callus (Heringer et al., 2015), however, its function is still poorly understood. Fumarylacetoacetate hydrolase domain (FH) exhibited the same pattern in CTC15 and SP90-3414, accumulating in response to drought. FH is a main enzyme acting in the final step of tyrosine (Tyr) degradation pathway (Shao et al., 2015). FH accumulated in leaves of maize seedlings (Shao et al., 2015) and it was reported to be involved with cell death in Arabidopsis (Han et al., 2013). It is possible that in sugarcane leaves, FH has a role acting in cell death, under drought.

### Metabolomic Responses of Sugarcane Leaves Under Drought

The metabolomic data also suggests a high plasticity of the sugarcane leaf metabolome, as a strategy to adjust its metabolism under drought. In terms of differentially abundant metabolites, a higher number was found in SP90-3414, this result may suggest that drought impact a different set of metabolites, in each variety and, the susceptible genotype shows the participation of a higher number of metabolites to face drought. This could also be a metabolic evidence of specific variety response, to drought. In accordance with this, only three differentially abundant metabolites [2-(4-hydroxyphenyl)-Ethanol, Hexadecane and taurine] were found in both varieties. The role of 2-(4-hydroxyphenyl)-Ethanol in plants still unknown. It was isolated from *Papaver rhoeas* leaves (Hillenbrand et al., 2004). Since this metabolite accumulated at 12DI, in both varieties, this phenolic compound could be involved in degradation or being the product of degradation, due to drought. Hexadecane was reported in pepper species (Kevrešan et al., 2013; Bogusz Junior et al., 2015). In our work, this alkane accumulated at PI and decrease under drought, indicating that either this metabolite or its precursors are degraded in response to drought. Taurine is an amino acid, known to act as osmolyte, accumulating in response to drought, salt and extreme temperatures (Liang et al., 2013). However, in our work, taurine reduced under drought. About the specific responses of each variety to drought, the sugars threose and rhamnose were not found at 12DI, both accumulated at PI and reduced the abundance at 4DI, in the CTC15 variety.Threose is one of the products from the oxidative degradation ascorbate, an important oxidant (Shumilina et al., 2019). Contrary to our findings, this sugar was reported to accumulate in roots of alfafa plants submitted to alkali stress (Song et al., 2017) and accumulate under drought in leaves, stem, root, and root colar in *Caragana korshinskii*, which is described as a highly drought tolerant plant (Zhang et al., 2017). Rhanose is a pectic component that constitutes the primary cell walls (Tenhaken, 2015), its reduction under drought might indicate the loosening of cell wall polysaccharides aiming to maintain cell expansion. The metabolite similar to Isopropyl B- d-1-thiogalactopyranoside accumulated at 4DI and was not found at 12DI, this metabolite was identified based on its probable class. In SP90-3414, the metabolites threonine, digitoxose, similar to N-(3-aminopropyl)-Morpholine, 3-ureido-Propionic acid, 4-hydroxy-Benzoic acid, indole-3-acetaldehyde enol MP, and 2-(4-hydroxyphenyl)-Ethanol, were differentially abundant in all conditions. All of them accumulated at PI and decreased in response to drought. The role of most of these metabolites is poorly understood in plants. However, they belong to different classes and might be related to different biochemical process, indicating the complexity of sugarcane responses to drought. Similar to icosapentaenoic acid, beta-D-Fructofuranosyl-(2,1)-beta-D-Fructofuranose and galactose changed their abbundace only between PI and 12DI and did not changed in comparison to 4DI. The first two metabolites accumulated at 12DI while galatose reduced at 12DI, being strongly influenced by drought.

### rCCA Network Correlation

Here, we applied rCCA to explore correlation structures between metabolites and proteins, in two sugarcane varieties, contrasting in drought tolerance. We propose that rCCA can be used as a powerful approach to track the main changes in response to abiotic and biotic stress. It is important to mention that as well as for metabolomic correlation network and gene co-expression, metabolite-protein correlation is not always in agreement with known biochemical pathways (Fukushima et al., 2011).

The novelty in our network results is the fact that most proteins were correlated with metabolites whose function are unknown, meaning that based on the protein function in the plant metabolism, we can have some light in deciphering the biological role of such metabolites in response to drought. By this exploratory approach, networks revealed variety specific requirements to face drought. Besides, it was observed that leaf metabolism was adjusted in a different manner, in response to 4DI and 12DI. In the analysis CTC15-PI-4DI, the tolerant variety did not show major different with the control plants at 4 days of drought (**Suplementary Figure 4A**). For CTC15 PI-12DI, the biggest cluster is related to Hexadecanoic acid, also known as palmitic acid (PA). This lipid was unique at 12DI and is one of the major fatty acids forming virtually all, natural lipids, which serve either as lipid background for storage fats and oils, or hydrophobic matrix of cell membranes, or components of cuticle waxes and polymers (Sidorov et al., 2014). PA was reported to accumulate in wheat (Marček et al., 2019) and soybean (Mohamed and Latif, 2017) genotypes in response to drought. PA is positively correlated with proteins involved in different biological roles. NadH-quinone oxidoreductase (SCAGAM2126G09) and cytochrome c oxidase (SCSFLR2031H06) are involved in the electron transport chain (Melo et al., 2004; Mansilla et al., 2018). Two proteins found were related to DNA and RNA metabolism: Dead-box ATP-dependent RNA helicase (SCRUHR1077B02), that is related to replication, repair, recombination, transcription, translation, ribosome biogenesis and splicing (Tujeta and Tujeta, 2004) and polypyrimidine tract-binding protein (PTB) (SCCCCL4012D10) related to alternative splicing regulation (Rühl et al., 2012). The overexpression of dead-box ATP-dependent RNA helicase, in rice, was reported to regulate transcripts involved in the abiotic and oxidative stress responses and, therefore, it functions as a molecular switch in different signaling pathways leading to stress tolerance (Wang et al., 2016; Nawaz and Kang, 2019). The ml domain (MD-2-related lipid-recognition) protein, which is implicated in lipid recognition, is also part of this cluster. Cluster 2 is formed by three metabolites from different chemical classes. Two of them, 3-Methyl-2-oxopentanoic-acid (organic acid) and O-Benzoyl-beta-D-glucoside (glucoside) are positively correlated with all proteins in the cluster. However, the compound similar to inositol (alcohol) was correlated only with peroxidase 5-like (SCBFRT1072H11) and aldehyde dehydrogenase (SCJFLR1073D12), proteins involved in cell antioxidation (Pandey et al., 2017) and detoxification (Brocker et al., 2013). Despite the lack of information about these metabolites in plant metabolism, they correlated positively with all proteins in the cluster, which seems to be involved with cell protection (antioxidant, detoxification, and protein degradation) and photosynthesis efficiency, suggesting this cluster might have a protection role. Light-harvesting chlorophyll a/b-binding proteins (LHCB) (SCJFSB1014F03.b) increased in tolerant genotypes of *Zea mays* (Benesova et al., 2012), and *Malus domestica* (Zhou et al., 2015) when exposed to drought. The LHCBs have been predicted to modulatate ROS homeostasis (Xu et al., 2012) and this role probably requires other proteins that can cope with ROS, to protect cell metabolism. On the other hand, the proteins might suggest that the three metabolites may also be involved in cell protection.

Negative correlation was observed in cluster 3. Galactaric acid (unique at PI) and 17-beta-streadiol (not found at 12DI) were negatively correlated to wd40-like beta propeller repeat (WDR) (SCCCRZ3004G04) and dead-box ATP-dependent RNA helicase (SCUTRZ3071H01), while Galacturonic acid (not found at 12DI) positively correlated with dead-box ATP-dependent RNA helicase and negatively correlated to protoporphyrinogen chloroplastic (SCMCRZ3064A10).

Galactaric acid is an organic acid derived from galacturonic acid (GalA). Galactaric acid was reported to decrease by salinity in Arabidopsis (Sanchez et al., 2008) and to increase in plants exposed to elevated CO2, under heat stress, in grass (Yu et al., 2012). 17-beta-stradiol is a steroid, reported to be involved in plant growth and flowering (Janeczko and Skoczowski, 2005; Lindemann, 2015). Both metabolites correlated to proteins that have a role in DNA/RNA metabolism and scaffolding proteins. These proteins might be required to regulate important cellular processes by interacting with multiple proteins, under drought. One possibility is that, due to their role in ceullar metabolism, the metabolites levels are reduced. Another possibility is that the metabolites abundance decreased in response to drought and these two proteins are required be regulate important cellular processes by interacting with multiple proteins. GalA is an important constituent of cell wall (Bethke et al., 2014) and is an intermediate in the biosynthesis of the antioxidant ascorbic acid (AsA) (Agius et al., 2003). GalA accumulated under drought in wheat genotypes (Marček et al., 2019) and bermudagrass leaves (Du et al., 2012). Authors suggested that drought could lead leaves to oxidative stress, thus GalA increased to averting oxidative damage under severe water stress (Du et al., 2012). This metabolite has not changed significantly in CTC15; however, it is probably required to the same purpose in sugarcane leaves. GalA correlated positively with protoporphyrinogen, the abundance of this protein enhanced in salt-treated maize chloroplasts, the author suggested this increment would alleviate oxidative stress in salt-stressed maize (Zhao et al., 2013). Drought leads to oxidative stress and this could be the reason for this correlation, in our study. On the other hand, GalA correlates negatively with the dead-box ATP protein.

In SP90-3414, rCCA revelad a more complex regulatory process undergoing in the drought-susceptible variety, in comparison to the tolerant one. The PI-4DI cluster 1 represents the biggest cluster and involves positive and negative correlations. Adenine, indole-3-acetaldehyde (lAAld), and octadecanal correlated with the same eleven proteins (**Figure 9A**). The three metabolites act in different process; adenine is one of the major purines and play important role in plant metabolism, as it represents the major energy currency of the cell (Haferkamp et al., 2011). Sukrong et al. (2012), suggested that adenine might play a part in the signals that modulate responses to abiotic stress and plant growth. lAAld can be oxidezed to the auxin indole-3-acetic acid (IAA) and is related to the tryptophan metabolim (Tsurusaki et al., 1997). Octadecanal is a lipid, found in the cuticular wax of maize (Perera and Nicolau, 2007) and epicular wax of pear fruits (Wu et al., 2018). All metabolites were negatively correlated with two chloroplastic proteins: translocase and a protease. Besides, these metabolites correlated positively with proteins related to a variety of molecular processs, such as oxidative stress (glutathione-s-transferase), photosynthetic regulation (peroxisomal-2-hydroxy-acid-oxidase), proteins degradation (26s proteasome and proteosomal subunit), signaling (14-3-3), chromating organization (histone h1), proteins chaperoning (dnaj protein), and monolignol biosynthesis (cinnamyl alcohol dehydrogenase). Octadecanoic acid, also known as stearic acid, was reported to decrease in sunflower seeds (Petcu et al., 2001) and soybean (Mohamed and Latif, 2017) submitted to drought. But it was reported to increase in the leaves of a drought-susceptible oat variety, under drought (Sánchez-Martín et al., 2018). This metabolite was positively correlated with histone h2 and negatively correlated with histone h1, peroxisomal oxidase, proteasome, 14-3-3, glutathione-s-trasnferase, and cinnamyl-alcohol dehydrogenase. Besides the complexity of this cluster, it is clear that the same set of proteins regulates or is regulated, positively and negatively, by two different sets of metabolites, suggesting the metabolites might have completely different roles under drought. The analysis for SP90-3414 PI-12DI resulted in five clusters. Cluster 1 is the most complex, involving positive and negative correlation and six metabolites: probable steroid, 3-hydroxy-Indole, trans-3-hydroxy-Proline, probable amino acid derivative, allo-Threonine, and Digitoxose. As reported to the other cluster, this one refers to a different set of metabolites, correlated with a variety of proteins, some of them related to cellular protein modification, transport, translation, response to stress, and cellular process. Two purple acid phosphatase precursor (PAP) protoforms (SCEQLR1091F02 and SCCCLR1075F03) were found in cluster 1. Both were positively correlated to 3-hydroxy-Indole. However, SCEQLR1091F02 was also correlated to the probable steroid metabolite while, SCCCLR1075F03 was correlated to trans-3-hydroxy-proline (**Figure 9B**). PAP is an acid phosphatase (APase) and acts in phosphorus (P) scavenging and recycling under conditions of P deficiency (Liu et al., 2016). It is known that, drought decreases the concentration of nutrient, such as P in plant tissues (reviewed in Bista et al., 2018). The identification of both proteins in SP90-3414 PI-12DI network, suggest that, at this level of stress, the susceptible variety might be dealing with P deficiency and require PAPs. In wheat exposed to drought, the authors (Sharma and Kaur, 2008) found a significant increase in APase activity in leaves and grains of drought tolerant plant, however, no change was observed in the drought sensitive. Curiosly, the SP90-3414-PI-12DI cluster 3 is composed by the same metabolites and almost the same proteins, found in the SP90-3414-PI-4DI network, suggesting that adenine, lAAld, octadecanal, as well as the proteins present in the SP90-3414-PI-12DI cluster 3 might be strongly influenced or impacted by drought, in the sugarcane susceptible variety.

## Conclusion

In summary, the analysis of sugarcane leaves from two drought-tolerant contrasting varieties revealed the dynamics of proteome and metabolome in response to drought. Considering the physiologycal responses to drought, this was observed earlier in the drought-susceptible variety (SP90-3414), in comparisson to the tolerant one. Proteomics revealed that different biological process had a stronger impact in each variety (*e*.*g*. translation in CTC15, generation of precursor metabolites and energy in SP90-3414). The differentially abundant metabolites found, in response to drought, were in agreement with previous observations, and reveals the plasticity of metabolism under drought. rCCA network revealed the complexity of protein-metabolite/metabolite-protein regulation. In addition, we exemplify that rCCA, based on protein and metabolite data, can be a powerfull strategy to identify potential networks; assign biological conclusion on metabolite-protein/protein-metabolite regulation and elucidate biological function of predicted metabolites and proteins.

## Author Contributions

IB conceived of the study, carried out the experiments, analyzed the data and wrote the manuscript. TC assisted with metabolomics and proteomics studies. FM performed the rCCA and LF assisted with proteomics data processing. CL conceived of the study, participated in its design and coordination, and reviewed the manuscript. All authors approved the final manuscript.

## Conflict of Interest

The authors declare that the research was conducted in the absence of any commercial or financial relationships that could be construed as a potential conflict of interest.

## Supplementary Material

Supplementary Figure 1 Effects of drought on photosynthetic rate - A in the varieties CTC15 (drought-tolerant) and SP90-3414 (drought-susceptible). Comparison among permanently irrigated (PI), four days without irrigation (4DI) and twelve days without irrigation (12DI). Letters indicate differences significant by Tukey’s test at 5% probability level.

Supplementary Figure 2 Venn diagram showing the number of common and unique proteins in each variety. A) CTC15 (drought-tolerant). B) SP90-3414 (drought-susceptible).

Supplementary Figure 3 Venn diagram showing overlapping proteins between CTC-15 (drought-tolerant) and SP90-3414 (drought-susceptible) varieties. A) Venn diagram based on the differentially abundant (p ≤ 0.05) proteins. B) Venn digram based on the unique proteins found in each variety.

Supplementary Figure 4 Venn diagram showing the number of common and unique metabolites in each variety. A) CTC15 (drought-tolerant). B) SP90-3414 (drought-susceptible).

Supplementary Figure 5 Unit representation plots of the rCCA for the first 2 canonical variates for both varieties, CTC15 (drought-tolerant) and SP90-3414(drought-susceptible). A) CTC-PI-4DI. B) CTC-PI-12DI. C) SP-PI-4DI. D) SP-PI-12DI. PI = permanently irrigated. 4DI = four days without irrigation. 12DI = twelve days without irrigation 12DI.

Supplementary Figure 6 Correlation circle plots of the rCCA for the first 2 components for both varieties, CTC15 (drought-tolerant) and SP90-3414(drought-susceptible). A) CTC-PI-4DI. B) CTC-PI-12DI. C) SP-PI-4DI. D) SP-PI-12DI.

Supplementary Table 1 Proteins exclusively found in each treatment (PI = permanently irrigated, 4DI = four days without irrigation, 12DI = twelve days without irrigation 12DI) in each variety CTC15 (drought-tolerat) and SP90-3414 (drought-susceptible).

Supplementary Table 2 Proteins differentially abundant identified by one-way Anova (FDR adjusted p ≤ 0.05) in the drought-tolerant variety CTC15.

Supplementary Table 3 Proteins differentially abundant identified by one-way Anova (FDR adjusted p ≤ 0.05) in the drought-susceptible variety SP90-3414.

Supplementary Table 4 Tab 1 - Metabolites found in CTC15 (drought-tolerant) variety. Tab 2 – Metabolites differentially abundant identified by one-way Anova (FDR adjusted p ≤ 0.05) in the drought-tolerant variety CTC15.

Supplementary Table 5 Tab 1 - Metabolites found in SP90-3414 (drought-susceptible) variety. Tab 2 – Metabolites differentially abundant identified by one-way Anova (FDR adjusted p ≤ 0.05) in the drought-susceptible variety SP90-3414.

Supplementary Table 6 Descriptive information about the metabolite-protein Network correlation at threshould 0.99 to CTC15 (drought-tolerant variety).

Supplementary Table 7 Descriptive information about the metabolite-protein Network correlation at threshould 0.99 to SP90-3414 (drought-susceptible variety).

## References

[B1] Agius F. González-Lamothe R. Caballero J. I. Muñoz-Blanco J. Botella M. A. Valpuesta V. (2003). Engineering increased vitamin C levels in plants by overexpression of a D-galacturonic acid reductase. Nat. Biotechnol. 21, 177–181. 10.1038/nbt777 12524550

[B2] Aharoni A. Galili G. (2011). Metabolic engineering of the plant primary-secondary metabolism interface. Curr. Opin. Biotechnol. 22, 239–244. 10.1016/j.copbio.2010.11.004 21144730

[B3] Almeida C. M. A. Silva T. D. Malafaia C. B. Amaral D. O. J. Arruda D. R. S. de Brito G. G. (2013). Proteomic and physiological analysis of response to water deficit in sugarcane. Wudpecker J. Agric. Res. 2, 001–007.

[B4] Benesova M. Hola D. Fischer L. Jedelsky P. L. Hnilicka F. Wilhelmova N. (2012). The physiology and proteomics of drought tolerance in maize: Early stomatal closure as a cause of lower tolerance to short-term dehydration? PloS One 7, e38017. 10.1371/journal.pone.0038017 22719860 PMC3374823

[B5] Benjamin J. G. Nielsen D. C. (2006). Water deficit effects on root distribution of soybean, field pea and chickpea. Field Crops Res. 97, 248–253. 10.1016/j.fcr.2005.10.005

[B6] Bethke G. Grundman R. E. Sreekanta S. Truman W. Katagiri F. Glazebrook J. (2014). *Arabidopsis* pectin methylesterases contribute to immunity against Pseudomonas syringae. Plant Physiol. 164, 1093–1107. 10.1104/pp.113.227637 24367018 PMC3912082

[B7] Bista D. R. Heckathorn S. A. Jayawardena D. M. Mishra S. Boldt J. K. (2018). Effects of drought on nutrient uptake and the levels of nutrient-uptake proteins in roots of drought-sensitive and -tolerant grasses. Plants (Basel) 7, 28. 10.3390/plants7020028 PMC6027393 29601475

[B8] Bogusz Junior S. Março P. H. Valderrama P. Damasceno F. C. Aranda M. S. Zini C. A. (2015). Analysis of volatile compounds in-*Capsicum*-spp. by headspace solid-phase microextraction and GC x GC-TOFMS. Anal. Methods 7, 521–529. 10.1039/c4ay01455c

[B9] Bradford M. M. (1976). Rapid and sensitive method for the quantitation of microgram quantities of protein utilizing the principle of protein-dye binding. Anal. Biochem. 72, 248–254. 10.1016/0003-2697(76)90527-3 942051

[B10] Brocker C. Vasiliou M. Carpenter S. Carpenter C. Zhang Y. Wang X. (2013). Aldehyde dehydrogenase (ALDH) superfamily in plants: gene nomenclature and comparative genomics. Planta 237, 189–210. 10.1007/s00425-012-1749-0 23007552 PMC3536936

[B11] Charitou T. Bryan K. Lynn D. J. (2016). Using biological networks to integrate, visualize and analyze genomics data. Genet. Sel. Evol. 48, 27. 10.1186/s12711-016-0205-1 27036106 PMC4818439

[B12] Cia M. C. Guimaraes A. C. R. Medici L. O. Chabregas S. M. Azevedo R. A. (2012). Antioxidant responses to water deficit by drought-tolerant and -sensitive sugarcane varieties. Ann. Appl. Biol. 161, 313–324. 10.1111/j.1744-7348.2012.00575.x

[B13] Conesa A. Götz S. García-Gómez J. M. Terol J. Talón M. Robles M. (2005). Blast2GO: a universal tool for annotation, visualization and analysis in functional genomics research. Bioinformatics 21, 3674–3676. 10.1093/bioinformatics/bti610 16081474

[B14] Cuadros-Inostroza A. Caldana C. Redestig H. Kusano M. Lisec J. Peña-Cortés H. (2009). TargetSearch –a bioconductor package for the efficient preprocessing of GC-MS metabolite profiling data. BMC Bioinf. 10, 428. 10.1186/1471-2105-10-428 PMC3087348 20015393

[B15] D’Hont A. Grivet L. Feldmann P. Rao S. Berding N. Glaszmann J. C. (1996). Characterization of the double genome structure of modern sugarcane cultivars (Saccharum spp.) by molecular cytogenetics. Mol. Gen. Genet. 250, 405–413. 10.1007/BF02174028 8602157

[B16] D’Hont A. Souza G. M. Menossi M. Vincentz M. Van-Sluys M. A. Glaszmann J. C. (2008). “Sugarcane: a major source of sweetness, alcohol, and bio-energy,” in Plant genetics and genomics: crops and models. Eds. Moore P. H. Ray M. (New York: Springer), 483–513.

[B17] Du H. Wang Z. Yu W. Huang B. (2012). Metabolic responses of hybrid bermudagrass to short-term and long-term drought stress. Am. Soc Hortic. Sci. 137, 411–420. 10.21273/JASHS.137.6.411

[B18] Ferreira T. H. S. Tsunada M. S. Bassi D. Araújo P. Mattiello L. Guidelli G. V. (2017). Sugarcane water stress tolerance mechanisms and its implications on developing biotechnology solutions. Front. Plant Sci. 8, 1077. 10.3389/fpls.2017.01077 28690620 PMC5481406

[B19] Fukushima A. Kanaya S. Nishida K. (2011). Integrated network analysis and effective tools in plant systems biology. Front. Plant Sci. 5, 598. 10.3389/fpls.2014.00598 PMC4219401 25408696

[B20] Ghannoum O. (2009). C4 photosynthesis and water stress. Ann. Bot. 103, 635–644. 10.1093/aob/mcn093 18552367 PMC2707343

[B21] Gonzalez I. Déjean S. Martin P. G. P. Baccini A. (2008). CCA: an R package to extend canonical correlation analysis. J. Stat. Software 23, 1–14. 10.18637/jss.v023.i12

[B22] Gonzalez I. Cao K. A. Davis M. J. Déjean S. (2012). Visualising associations between paired ‘omics’ data sets. BioData Min. 5, 19. 10.1186/1756-0381-5-19 23148523 PMC3630015

[B23] Graça J. P. Rodrigues F. A. Farias J. R. B. Oliveira M. C. N. Hoffmann-Campo C. B. Zingaretti S. M. (2010). Physiological parameters in sugarcane cultivars submitted to water deficit. Braz. J. Plant Physiol. 22, 189–197. 10.1590/S1677-04202010000300006

[B24] Gullberg J. Jonsson P. Nordstrom A. Sjostrom M. Moritz T. (2004). Design of experiments: an efficient strategy to identify factors influencing extraction and derivatization of Arabidopsis thaliana samples in metabolomic studies with gas chromatography/mass spectrometry. Anal. Biochem. 331, 283–295. 10.1016/j.ab.2004.04.037 15265734

[B25] Haferkamp I. Fernie A. R. Neuhaus H. E. (2011). Adenine nucleotide transport in plants: much more than a mitochondrial issue. Trends Plant Sci. 16, 507–515. 10.1016/j.tplants.2011.04.001 21622019

[B26] Han C. Ren C. Zhi T. Zhou Z. Liu Y. Chen F. (2013). Disruption of fumarylacetoacetate hydrolase causes spontaneous cell death under short-day conditions in Arabidopsis. Plant Physiol. 162, 1956–1964. 10.1104/pp.113.216804 23743712 PMC3729774

[B27] Hand S. C. Menze M. A. Toner M. Boswell L. Moore D. (2011). LEA proteins during water stress: not just for plants anymore. Annu. Rev. Physiol. 73, 115–134. 10.1146/annurev-physiol-012110-142203 21034219

[B28] Heringer A. S. Barroso T. Macedo A. F. Santa-Catarina C. Souza G. H. Floh E. I. (2015). Label-free quantitative proteomics of embryogenic and non-embryogenic callus during sugarcane somatic embryogenesis. PloS One 10, e0127803. 10.1371/journal.pone.0127803 26035435 PMC4452777

[B29] Hillenbrand M. Zapp J. Becker H. (2004). Depsides from the Petals of Papaver rhoeas. Planta Med. 70, 380–382. 10.1055/s-2004-818956 15095160

[B30] Hoang N. V. Furtado A. Botha F. C. Simmons B. L. Henry R. J. (2015). Potential for genetic improvement of sugarcane as a source of biomass for biofuels. Front. Bioeng. Biotechnol. 3, 182. 10.3389/fbioe.2015.00182 26636072 PMC4646955

[B31] Hurkman W. J. Tanaka C. K. (1986). Solubilization of plant membrane proteins for analysis by two-dimensional gel electrophoresis. Plant Physiol. 81, 802–806. 10.1104/pp.81.3.802 16664906 PMC1075430

[B32] Jain R. Chandra A. Venugopalan V. K. Solomon S. (2015). Physiological changes and expression of SOD and P5CS genes in response to water deficit in sugarcane. Sugar Tech. 17, 276–282. 10.1007/s12355-014-0317-2

[B33] Janeczko A. Skoczowski A. (2005). Mammalian sex hormones in plants. Folia Histochem Cytobiol. 43, 70–71. 16044944

[B34] Jangpromma N. Songsri P. Thammasirirak S. Jaisil P. (2010). Rapid assessment of chlorophyll content in sugarcane using a SPAD chlorophyll meter across different water stress conditions. Asian J. Plant Sci. 9, 368–374. 10.3923/ajps.2010.368.374

[B35] Kevrešan Z. S. Mastilovic J. S. Sinadinovic-Fiser S. Hrabovski N. C. Radusin T. I. (2013). Spice paprika volatiles as affected by different postharvest ripening treatments of red pepper (Capsicum annuum L.) variety Aleva NK. Acta Period. Technol. APTEFF 44, 75–86. 10.2298/APT1344075K

[B36] Khueychai S. Jangpromma N. Daduang S. Jaisil P. Lomthaisong K. Dhiravisit A. (2015). Comparative proteomic analysis of leaves, leaf sheaths, and roots of drought-contrasting sugarcane cultivars in response to drought stress. Acta Physiol. Plant 37, 88. 10.1007/s11738-015-1826-7

[B37] Kido E. A. Neto J. R. C. F. de Oliveira Silva R. L. Pandolfi V. Guimaraes A. C. R. Veiga D. T. (2012). New insights in the sugarcane transcriptome responding to drought stress as revealed by supersage. Sci. World J. 2012, 821062. 10.1100/2012/821062 PMC3353566 22629208

[B38] Kopka J. Schauer N. Krueger S. Birkemeyer C. Usadel B. Bergmuller E. (2005). GMD@CSBDB: the Golm Metabolome Database. Bioinformatics 21, 1635–1638. 10.1093/bioinformatics/bti236 15613389

[B39] Lee H. Hsu K. Sajdak J. Qin J. Pavlidis P. (2004). Coexpression analysis of human genes across many microarray datasets. Genome Res. 14, 1085–1094. 10.1101/gr.1910904 15173114 PMC419787

[B40] Li C. Nong Q. Solanki M. K. Liang Q. Xie J. Liu X. (2016). Differential expression profiles and pathways of genes in sugarcane leaf at elongation stage in response to drought stress. Sci. Rep. 6, 25698. 10.1038/srep25698 27170459 PMC4864372

[B41] Liang X. Zhang L. Natarajan S. K. Becker D. F. (2013). Proline mechanisms of stress survival. Antioxid. Redox Signal. 19, 998–1011. 10.1089/ars.2012.5074 23581681 PMC3763223

[B42] Lindemann P. (2015). Steroidogenesis in plants – Biosynthesis and conversions of progesterone and other pregnane derivatives. Steroids 103, 145–152. 10.1016/j.steroids.2015.08.007 26282543

[B43] Liu P. D. Xue Y. B. Chen Z. J. Liu G. D. Tian J. (2016). Characterization of purple acid phosphatases involved in extracellular dNTP utilization in Stylosanthes. J. Exp. Bot. 67, 4141–4154. 10.1093/jxb/erw190 27194738 PMC5301924

[B44] Mansilla N. Racca S. Gras D. Gonzalez D. Welchen E. (2018). The complexity of mitochondrial Complex IV: an update of cytochrome c oxidase biogenesis in Plants. Int. J. Mol. Sci. 19, 662. 10.3390/ijms19030662 PMC5877523 29495437

[B45] Marček T. Hamow K.Á. Végh B. Janda T. Darko E. (2019). Metabolic response to drought in six winter wheat genotypes. PloS One 14 (2), e0212411. 10.1371/journal.pone.0212411 30779775 PMC6380608

[B46] Melo A. M. Bandeiras T. M. Teixeira M. (2004). New insights into type II NAD(P)H:quinone oxidoreductases Microbiol. Mol. Biol. Rev. 68, 603–616. 10.1016/j.protis.2018.11.001 PMC539002 15590775

[B47] Merewitz E. B. Gianfagna T. Huang B. (2011). Protein accumulation in leaves and roots associated with improved drought tolerance in creeping bentgrass expressing an ipt gene for cytokinin synthesis. J. Exp. Bot. 62, 5311–5333. 10.1093/jxb/err166 21831843 PMC3223035

[B48] Mohamed H. I. Latif H. H. (2017). Improvement of drought tolerance of soybean plants by using methyl jasmonate. Physiol. Mol. Biol. Plants 23 (3), 545–556. 10.1007/s12298-017-0451-x 28878493 PMC5567712

[B49] Montastier E. Villa-Vialaneix N. Caspar-Bauguil S. Hlavaty P. Tvrzicka E. Gonzalez I. (2015). System model network for adipose tissue signatures related to weight changes in response to calorie restriction and subsequent weight maintenance. PloS Comput. Biol. 11, e1004047. 10.1371/journal.pcbi.1004047 25590576 PMC4295881

[B50] Nawaz G. Kang H. (2019). Rice OsRH58, a chloroplast DEAD-box RNA helicase, improves salt or drought stress tolerance in Arabidopsis by affecting chloroplast translation. BMC Plant Biol. 19, 1–11. 10.1186/s12870-018-1623-8 30626336 PMC6327599

[B51] Naylor S. Culbertson A. W. Valentine S. J. (2008). Towards a systems level analysis of health and nutrition. Curr. Opin. Biotechnol. 19, 100–109. 10.1016/j.copbio.2008.02.009 18387294

[B52] Nouri M. Moumeni A. Komatsu S. (2015). Abiotic stresses: insight into gene regulation and protein expression in photosynthetic pathways of plants. Int. J. Mol. Sci. 16, 20392–20416. 10.3390/ijms160920392 26343644 PMC4613210

[B53] Pandey V. P. Awasthi M. Singh S. Tiwari S. Dwivedi U. N. (2017). A comprehensive review on function and application of plant peroxidases. Biochem. Anal. Biochem. 6, 1. 10.4172/2161-1009.1000308

[B54] Perera M. A. D. N. Nikolau B. J. (2007). Metabolomics of cuticular waxes: aa system for metabolomics analysis of a single tissue-type in a multicellular organism. In Concepts in Plant Metabolomics. Eds. Nikolau B. J. E. S. Wurtele (Dordrecht: Springer). 10.1007/978-1-4020-5608-6_8

[B55] Petcu E. Arsintesscu A. Stanciu D. (2001). The effect of drought stress on fatty acids composition in some Romanian sunflower hybrids. Rom. Agric. Res. 15, 39–43.

[B56] Ramesh P. (2000). Effect of different levels of drought during the formative phase on growth parameters and its relationship with dry matter accumulation in sugarcane. J. Agron. Crop Sci. 185, 83–89. 10.1046/j.1439-037x.2000.00404.x

[B57] Rodrigues F. A. Laia M. L. Zingaretti S. M. (2009). Analysis of gene expression profiles under water stress in tolerant and sensitive sugarcane plant. Plant Sci. 176, 286–302. 10.1016/j.plantsci.2008.11.007

[B58] Rodrigues – Rodrigues F. A. Da Graça J. P. De Laia M. L. Nhani-Jr A. Galbiati J. A. Ferro M. I. T. Ferro J. A. (2011). Sugarcane genes differentially expressed during water deficit. Biol. Plant 55, 43–53. 10.1007/s10535-011-0006-x

[B59] Rohart F. Gautier B. Singh A. Lê Cao K A. (2017) mixOmics: An R package for ‘omics feature selection and multiple data integration. PLoS Comput Biol 13 (11), e1005752. 10.1371/journal.pcbi.1005752 29099853 PMC5687754

[B60] Rühl C. Stauffer E. Kahles A. Wagner G. Drechsel G. Rätsch G. (2012). Polypyrimidine tract binding protein homologs from Arabidopsis are key regulators of alternative splicing with implications in fundamental developmental processes. Plant Cell. 24, 4360–4375. 10.1105/tpc.112.103622 23192226 PMC3531839

[B61] Sánchez-Martín J. Canales F. J. Tweed J. K. S. Lee M. R. F. Rubiales D. Gómez-Cadenas A. (2018). Fatty acid profile changes during gradual soil water depletion in oats suggests a role for jasmonates in coping with drought. Front. Plant Sci. 9, 1077. 10.3389/fpls.2018.01077 30131815 PMC6090161

[B62] Sanchez D. H. Siahpoosh M. R. Roessner U. Udvardi M. Kopka J. (2008). Plant metabolomics reveals conserved and divergent metabolic responses to salinity. Physiol. Plant 132, 209–219. 10.1111/j.1399-3054.2007.00993.x 18251862

[B63] Schauer N. Steinhauser D. Strelkov S. Schomburg D. Allison G. Moritz T. (2005). GC-MS libraries for the rapid identification of metabolites in complex biological samples. FEBS Lett. 579, 1332–1337. 10.1016/j.febslet.2005.01.029 15733837

[B64] Shannon P. Markiel A. Ozier O. Baliga N. S. Wang J. T. Ramage D. (2003). Cytoscape: a software environment for integrated models of biomolecular interaction networks. Genome Res. 13-11, 2498–2504. 10.1101/gr.1239303 PMC403769 14597658

[B65] Shao R. Xin L. Mao J. Li L. Kang G. Yang Q. (2015). Physiological, ultrastructural and proteomic responses in the leaf of maize seedlings to polyethylene glycol-stimulated severe water deficiency. Int. J. Mol. Sci. 16, 21606–21625. 10.3390/ijms160921606 26370980 PMC4613270

[B66] Sharma A. D. Kaur P. (2008). Drought-stress induced changes in the expression of acid phosphatases in drought tolerant and susceptible cultivars of wheat. World J. Agric. Sci. 4, 471–475.

[B67] Shumilina J. Kusnetsova A. Tsarev A. Janse van Rensburg H. C. Medvedev S. Demidchik V. (2019). Glycation of plant proteins: regulatory roles and interplay with sugar signalling? Int. J. Mol. Sci. 20, 2366. 10.3390/ijms20092366 PMC6539852 31086058

[B68] Sidorov R. A. Zhukov A. V. Pchelkin V. P. Tsydendambaev V. (2014). “Palmitic acid in higher plant lipids,” in Palmitic Acid: Occurrence, Biochemistry and Health Effects, Chapter: 6. Ed. Porto Lukas F (New York: Nova Science Publishers). 125–143.

[B69] Silva J. C. Gorenstein M. V. Li G. Z. Vissers J. P. Geromanos S. J. (2006). Absolute quantification of proteins by LCMSE: a virtue of parallel MS acquisition. Mol. Cell. Proteomics 5, 144–156. 10.1074/mcp.M500230-MCP200 16219938

[B70] Silva M. D. A. Jifon J. L. dos Santos C. M. Jadoski C. J. da Silva J. A. G. (2013). Photosynthetic capacity and water use efficiency in sugarcane genotypes subject to water deficit during early growth phase. Braz. Arch. Biol. Technol. 56, 735–748. 10.1590/S1516-89132013000500004

[B71] Singh S. Rao P. N. G. (1987). Varietal differences in growth characteristics in sugarcane. J. Agric. Sci. 108, 245–247. 10.1017/S0021859600064327

[B72] Song T. Xu H. Sun N. Jiang L. Tian P. Yong Y. (2017). Metabolomic analysis of alfalfa (*Medicago sativa*-L.) root-symbiotic rhizobia responses under alkali stress. Front. In Plant Sci. 8, 1208. 10.3389/fpls.2017.01208 28744296 PMC5504246

[B73] Sukrong S. Yun K. Y. Stadler P. Kumar C. Facciuolo T. Moffatt B. A. (2012). Improved growth and stress tolerance in the Arabidopsis oxt1 mutant triggered by altered adenine metabolism. Mol. Plant 5, 1310–1332. 10.1093/mp/sss065 22859732

[B74] Tenhaken R. (2015). Cell wall remodeling under abiotic stress. Front. Plant Sci. 5, 771. 10.3389/fpls.2014.00771 25709610 PMC4285730

[B75] Thiebaut F. Grativol C. Tanurdzic M. Carnavale-Bottino M. Vieira T. Motta M. R. (2014). Differential sRNA regulation in leaves and roots of sugarcane under water depletion. PloS One 9, e93822. 10.1371/journal.pone.0093822 24695493 PMC3973653

[B76] Tsurusaki K. K. Takeda K. Sakurai K. (1997). Conversion of Indole-3-Acetaldehyde to Indole-3-Acetic acid in cell-wall fraction of Barley (Hordeum vulgare) Seedlings. Plant Cell Physiol. 38, 268–273. 10.1093/oxfordjournals.pcp.a029162

[B77] Tuteja N. Tuteja R. (2004). Unraveling DNA helicases. Motif, structure, mechanism and function. Eur. J. Biochem. 271, 1849–1863. 10.1111/j.1432-1033.2004.04094.x 15128295

[B78] Vantini J. S. Dedemo G. C. Jovino Gimenez D. F. Fonseca L. F. Tezza R. I. Mutton M. A. (2015). Differential gene expression in drought-tolerant sugarcane roots. Genet. Mol. Res. 14, 7196–7207. 10.4238/2015.June.29.13 26125930

[B79] Vargas L. Santa Brígida A. B. Mota Filho J. P. de Carvalho T. G. Rojas C. A. Vaneechoutte D. (2014). Drought tolerance conferred to sugarcane by association with gluconacetobacter diazotrophicus: a transcriptomic view of hormone pathways. PloS One 9 (12), e114744. 10.1371/journal.pone.0114744 25489849 PMC4260876

[B80] Vettore A. L. da Silva F. R. Kemper E. L. da Silva A. M. Ferro M. I. (2003). Analysis and functional annotation of an expressed sequence tag collection for tropical crop sugarcane. Genome Res. 13, 2725–2735. 10.1101/gr.1532103 14613979 PMC403815

[B81] Vizcaíno J. A. Csordas A. del-Toro N. Dianes J. A. Griss J. Lavidas I. (2016). Update of the PRIDE database and related tools. Nucleic Acids Res. 44, D447–D456. 10.1093/nar/gkv1145 26527722 PMC4702828

[B82] Wahid A. Close T. J. (2007). Expression of dehydrins under heat stress and their relationship with water relations of sugarcane leaves. Biol. Plant 51, 104–109. 10.1007/s10535-007-0021-0

[B83] Wan X. Y. Liu J. Y. (2008). Comparative proteomics analysis reveals an intimate protein network provoked by hydrogen peroxide stress in rice seedling leaves. Mol. Cell. Proteomics 7, 1469–1488. 10.1074/mcp.M700488-MCP200 18407957 PMC2500226

[B84] Wang W. Vinocur B. Altman A. (2003). -Plant responses to drought, salinity and extreme temperatures: towards genetic engineering for stress tolerance. Planta 218, 1–14. 10.1007/s00425-003-1105-5 14513379

[B85] Wang D. Qin B. Li X. Tang D. Zhang Y. Cheng Z. (2016). Nucleolar DEAD-box RNA helicase TOGR1 regulates thermotolerant growth as a pre-rRNA chaperone in rice. PloS Genet. 12 (2), e1005844. 10.1371/journal.pgen.1005844 26848586 PMC4743921

[B86] Wu X. Yin H. Shi Z. Chen Y. Qi K. Qiao X. (2018). -Chemical composition and crystal morphology of epicuticular wax in mature fruits of 35 pear (*Pyrus*-spp.) cultivars. Front. Plant Sci. 9, 679. 10.3389/fpls.2018.00679 29875784 PMC5974152

[B87] Xia J. Sinelnikov I. V. Han B. Wishart D. S. (2015). MetaboAnalyst 3.0-making metabolomics more meaningful. Nucleic Acids Res. 43, W251–W257. 10.1093/nar/gkv380 25897128 PMC4489235

[B88] Xu Y. H. Liu R. Yan L. Liu Z. Q. Jiang S. C. Shen Y. Y. (2012). Light-harvesting chlorophyll a/b-binding proteins are required for stomatal response to abscisic acid in Arabidopsis. J. Exp. Bot. 63, 1095–1106. 10.1093/jxb/err315 22143917 PMC3276081

[B89] Yu J. Du H. Xu M. Huang B. (2012). Metabolic responses to heat stress under elevated atmospheric co2 concentration in a cool-season grass species. Am. Soc Hortic. Sci. 137, 221–228. 10.21273/JASHS.137.4.221

[B90] Zhang J. Chen G. Zhao P. Zhou Q. Zhao X. (2017). The abundance of certain metabolites responds to drought stress in the highly drought tolerant plant Caragana korshinskii. Acta Physiol. Plant 39, 116. 10.1007/s11738-017-2412-y

[B91] Zhao D. Glaz B. Comstock J. C. (2013). Sugarcane leaf photosynthesis and growth characters during development of water-deficit stress. Crop Sci. 53, 1066–1075. 10.2135/cropsci2012.09.0554

[B92] Zhou G. Yang L. T. Li Y. R. Zou C. L. Huang L. P. Qiu L. H. (2012). Proteomic analysis of osmotic stress responsive proteins in sugarcane leaves. Plant Mol. Biol. Rep. 30, 349–359. 10.1007/s11105-011-0343-0

[B93] Zhou S. Li M. Guan Q. Liu F. Zhang S. Chen W. (2015). Physiological and proteome analysis suggest critical roles for the photosynthetic system for high water-use efficiency under drought stress in Malus. Plant Sci. 236, 44–60. 10.1016/j.plantsci.2015.03.017 26025520

[B94] Zingaretti M. Z. Rodrigues F. A. Graça J. P. Pereira L. M. Lourenço M. V. (2012). “Sugarcane Responses at Water Deficit Conditions,” in Water Stress. Ed. Rahman I. Md. M. (Rijeka, Croatia: Intech), 255–276. 10.5772/30986

