# Supplementary figures and images for: Network Analyses and Data Integration of Proteomics and Metabolomics From Leaves of Two Contrasting Varieties of Sugarcane in Response to Drought
Source: Front Plant Sci. 2019 Nov 28;10:1524. doi: 10.3389/fpls.2019.01524 (PMC6892781; doi:10.3389/fpls.2019.01524)

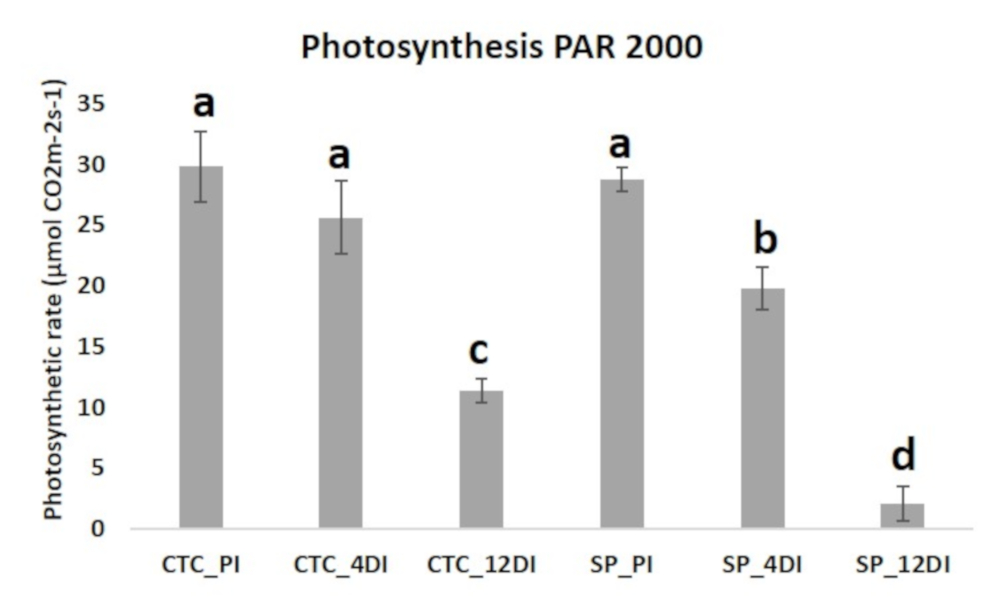

Supplement: Supplementary Figure 1 — Effects of drought on photosynthetic rate - A in the varieties CTC15 (drought-tolerant) and SP90-3414 (drought-susceptible). Comparison among permanently irrigated (PI), four days without irrigation (4DI) and twelve days without irrigation (12DI). Letters indicate differences significant by Tukey’s test at 5% probability level. [file Image_1.jpeg]

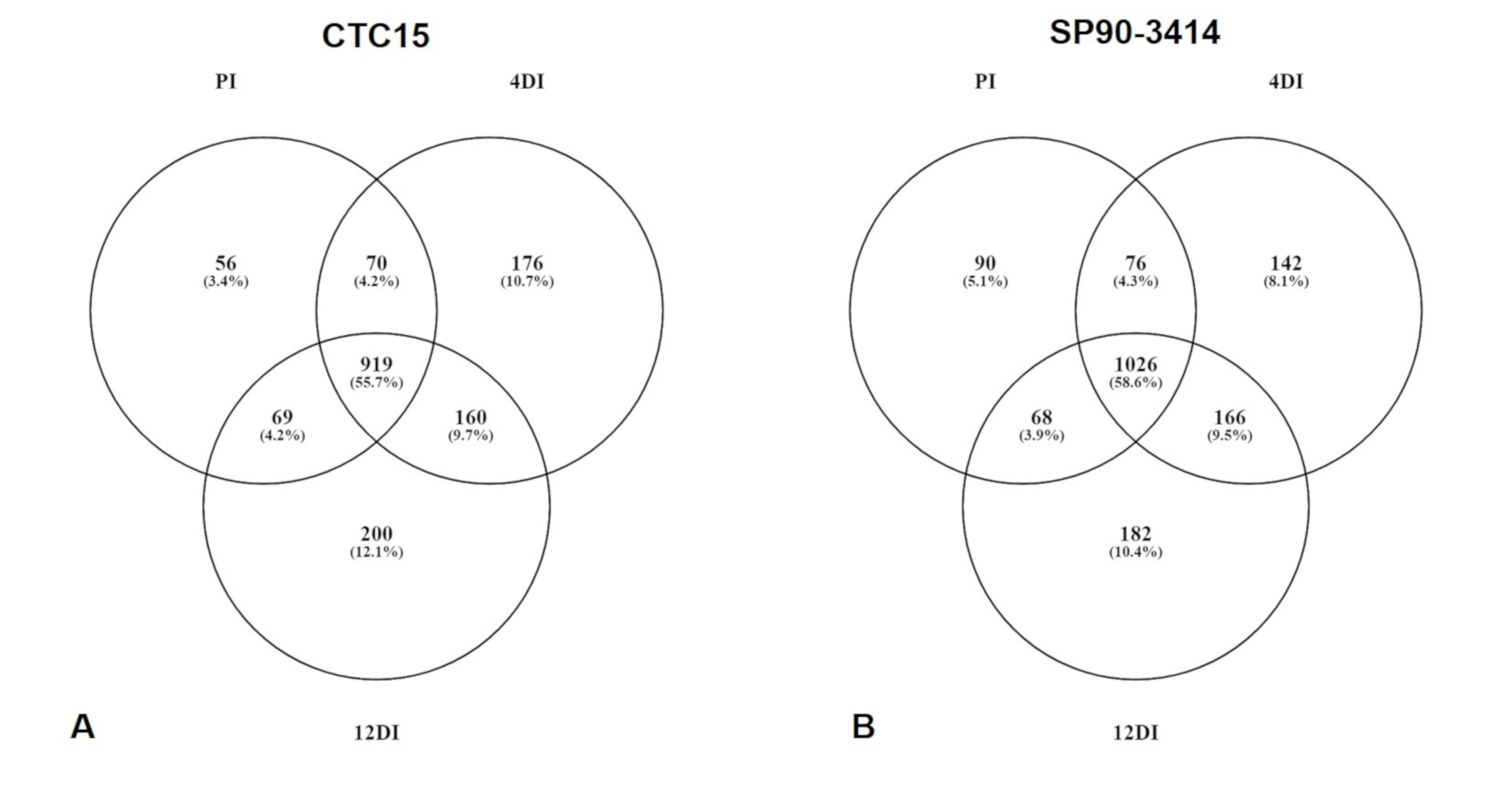

Supplement: Supplementary Figure 2 — Venn diagram showing the number of common and unique proteins in each variety. A) CTC15 (drought-tolerant). B) SP90-3414 (drought-susceptible). [file Image_2.jpeg]

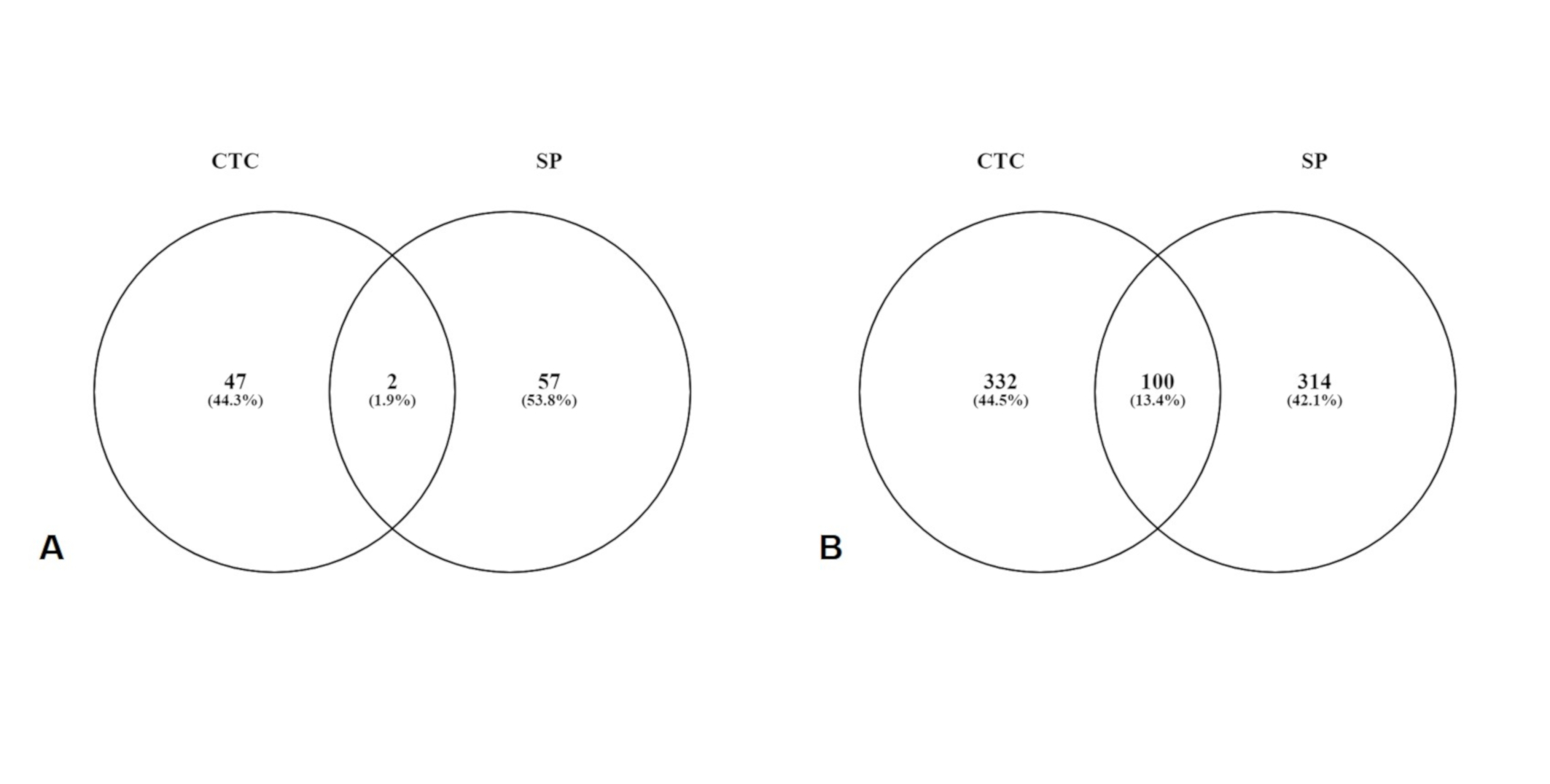

Supplement: Supplementary Figure 3 — Venn diagram showing overlapping proteins between CTC-15 (drought-tolerant) and SP90-3414 (drought-susceptible) varieties. A) Venn diagram based on the differentially abundant (p ≤ 0.05) proteins. B) Venn digram based on the unique proteins found in each variety. [file Image_3.jpeg]

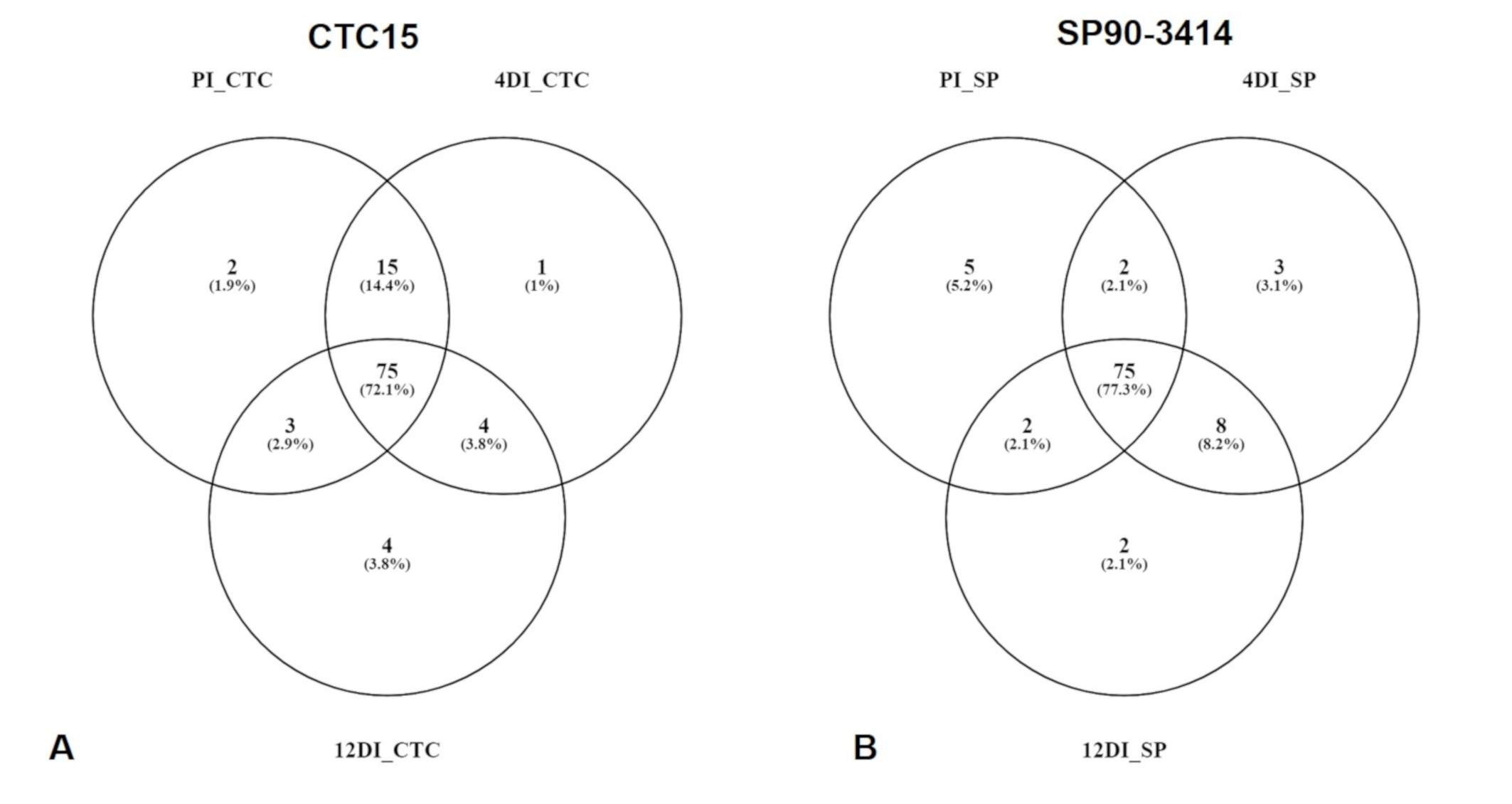

Supplement: Supplementary Figure 4 — Venn diagram showing the number of common and unique metabolites in each variety. A) CTC15 (drought-tolerant). B) SP90-3414 (drought-susceptible). [file Image_4.jpeg]

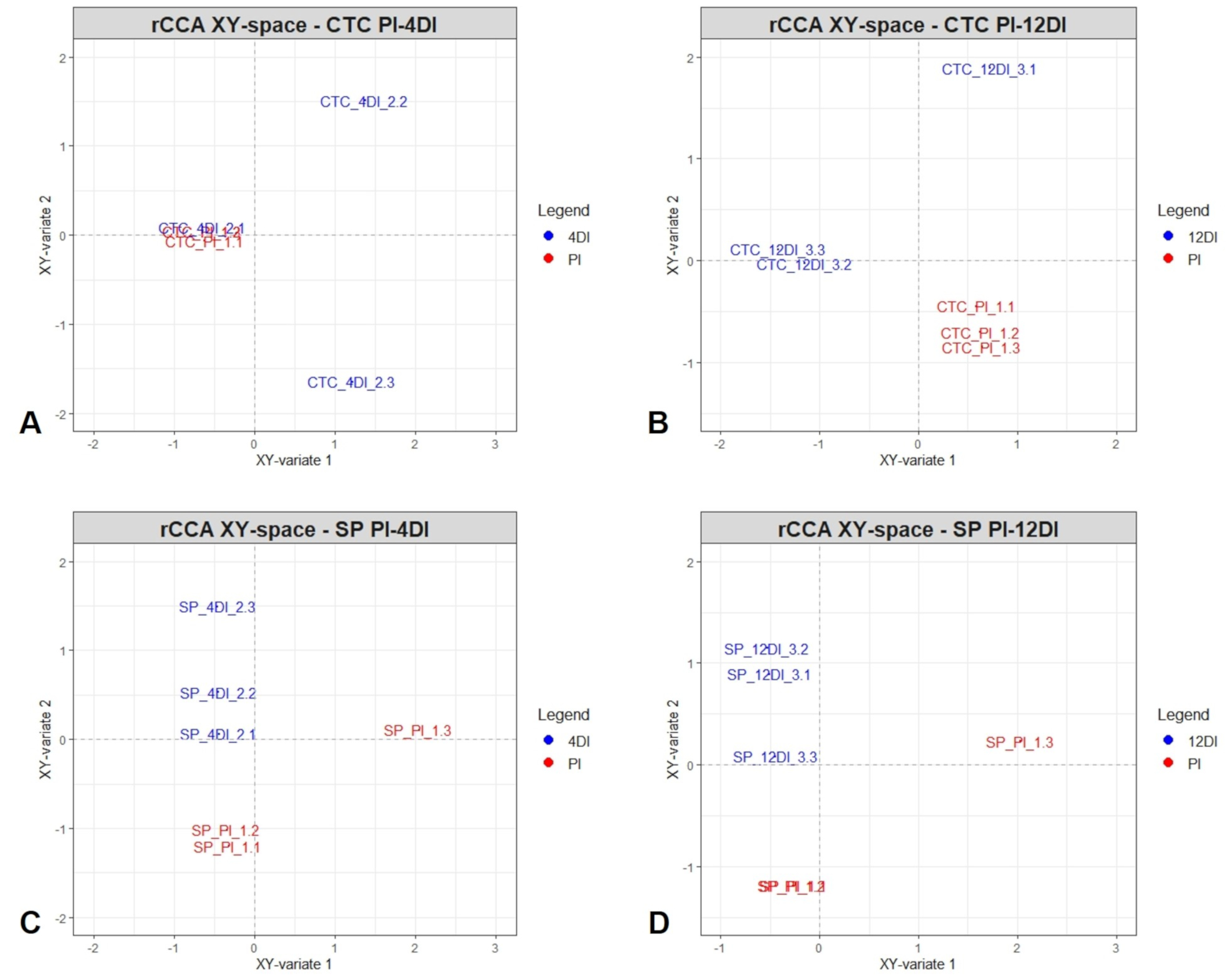

Supplement: Supplementary Figure 5 — Unit representation plots of the rCCA for the first 2 canonical variates for both varieties, CTC15 (drought-tolerant) and SP90-3414(drought-susceptible). A) CTC-PI-4DI. B) CTC-PI-12DI. C) SP-PI-4DI. D) SP-PI-12DI. PI = permanently irrigated. 4DI = four days without irrigation. 12DI = twelve days without irrigation 12DI. [file Image_5.jpeg]

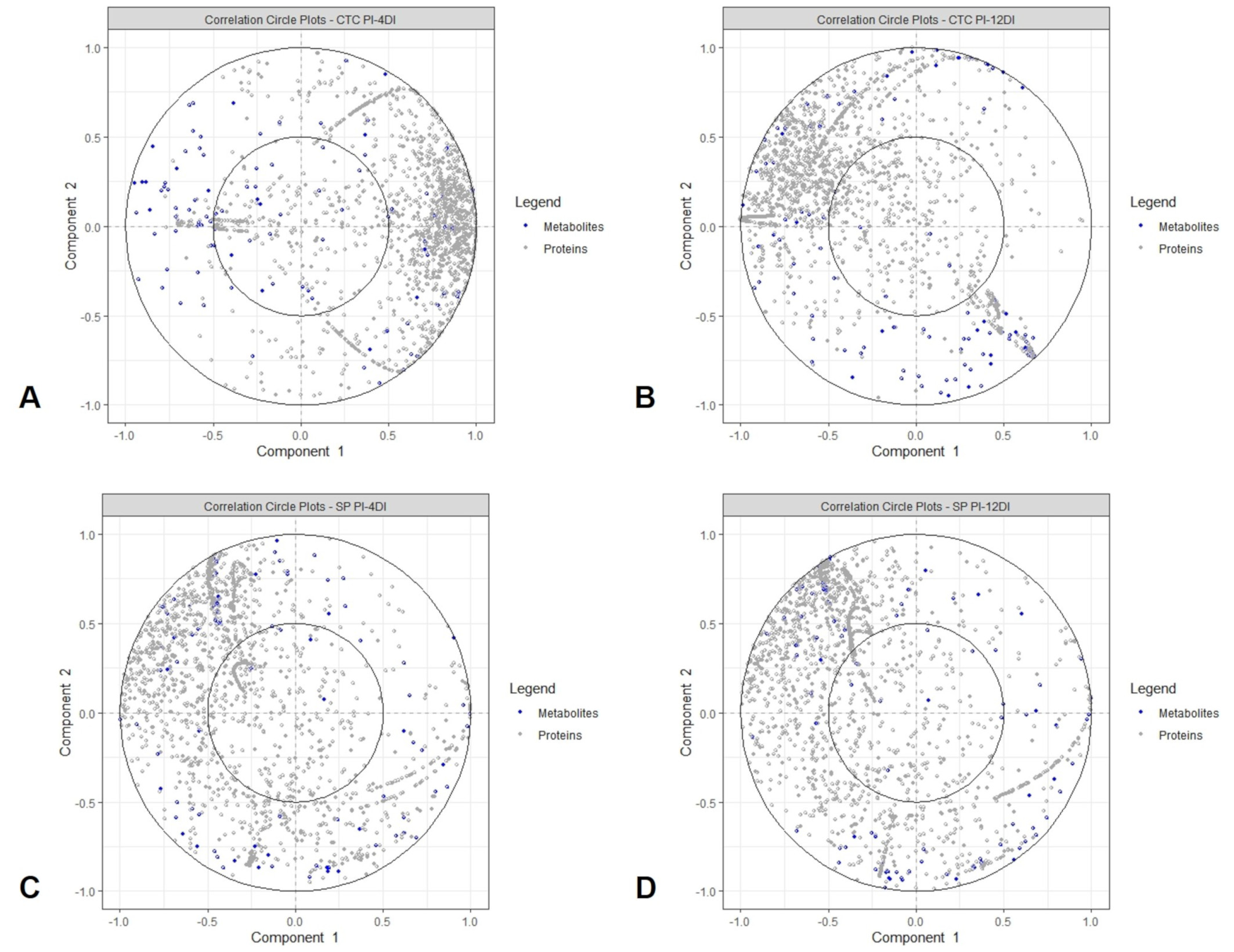

Supplement: Supplementary Figure 6 — Correlation circle plots of the rCCA for the first 2 components for both varieties, CTC15 (drought-tolerant) and SP90-3414(drought-susceptible). A) CTC-PI-4DI. B) CTC-PI-12DI. C) SP-PI-4DI. D) SP-PI-12DI. [file Image_6.jpeg]
